# Heart failure induced by isoproterenol: A comparison of two doses and two delivery methods in C57BL/6J mice

**DOI:** 10.1371/journal.pone.0334880

**Published:** 2025-11-03

**Authors:** Yaojiang Wang, Rong Yang, Aonan Yu, Peng Yang, Shaojie Huang, Yuqing Ye, Chunxiao Zhu, Mengxiao Zhang

**Affiliations:** 1 School of Pharmacy, Bengbu Medical University, Bengbu, China; 2 School of Stomatology, Bengbu Medical University, Bengbu, China; 3 Anhui Engineering Technology Research Center of Biochemical Pharmaceutical, Bengbu Medical University, Bengbu, China; Tokyo Women's Medical University, JAPAN

## Abstract

Heart failure (HF) modeling requires standardized protocols to ensure translational relevance. Despite the widespread use of isoproterenol (ISO)—a β-adrenergic agonist—in HF modeling, methodological inconsistencies in dosing and administration routes limit reproducibility. This study evaluated the effects of subcutaneous (SC) and intraperitoneal (IP) administration of ISO at two literature-established doses (5 and 60 mg/kg/day for 14 days) on cardiac remodeling in C57BL/6J mice, aiming to identify the optimal protocol for HF modeling. Using a factorial design, male C57BL/6J mice aged 6–8 weeks were divided into six cohorts: (1) SC saline control, (2) IP saline control, (3) SC 5 mg/kg ISO, (4) IP 5 mg/kg ISO, (5) SC 60 mg/kg ISO, and (6) IP 60 mg/kg ISO, with daily administration for 14 days. High-dose ISO (60 mg/kg/day) induced a 25% mortality rate in both SC and IP cohorts, yet IP administration exhibited marked inter-individual variability, undermining model reliability. Echocardiography revealed SC 5 mg/kg group induced stable systolic dysfunction accompanied by left ventricular dilation, while maintaining 100% survival. This cohort also displayed significantly elevated hypertrophy indices. Histopathological quantification suggested that SC 60 mg/kg induced extensive fibrosis. All ISO-treated groups showed upregulated myocardial hypertrophy markers and approximately 2-fold elevation in serum NT-proBNP levels. In summary, SC 5 mg/kg/day regimen not only ensures reliable phenotype induction but also reduces animal attrition, offering a robust platform for investigating CHF mechanisms and accelerating therapeutic development.

## Introduction

Heart failure (HF) is a syndrome in which a variety of factors affect the structure and function of the heart, leading to impaired ventricular filling and/or ejection. This results in deficient cardiac output that fails to meet the basic metabolic needs of systemic tissues and represents the end stage of a variety of heart diseases [1,2]. The annual increase in HF morbidity and mortality is now a major challenge in HF prevention and control [3–5]. Due to the diversity of HF types and the wide variation in patient individualization, many patients progress to the stage of advanced HF when symptoms persist despite maximal treatment [6]. Consequently, it is of great significance to study the pathological changes and corresponding mechanisms of chronic HF. The preparation of stable and reliable animal models with similar characteristics to human clinical conditions is the key to experimentation [7].

Currently, common mouse models of HF include aortic constriction models, coronary artery ligation models, and drug-induced HF models [8]. Isoproterenol (ISO), a non-selective β-adrenergic agonist, mimics the neurohumoral overactivation in HF by stimulating cardiac G-protein-coupled β-adrenergic receptors, causing cardiomyocyte hypertrophy and myocardial fibrosis [9]. ISO-induced HF modeling is easy to operate and has become one of the most commonly used models in HF research [10]. However, published studies reveal significant variability regarding ISO doses and administration methods used in the preparation of HF models, which has hampered HF-related research.

To address this, the present study compared low and high doses of ISO (5 *vs.* 60 mg/kg/day) delivered via both subcutaneous and intraperitoneal routes in C57BL/6J mice. These doses represent well-established extremes able to elicit distinct phenotypic responses, thereby enabling clear evaluation of dose- and administration-dependent effects. This study aims to identify a robust and standardized protocol for ISO-induced HF modeling to support future mechanistic and therapeutic investigations.

## Results

### Impact of different modeling protocol on survival of C57BL/6J mice

**Fig 1:**
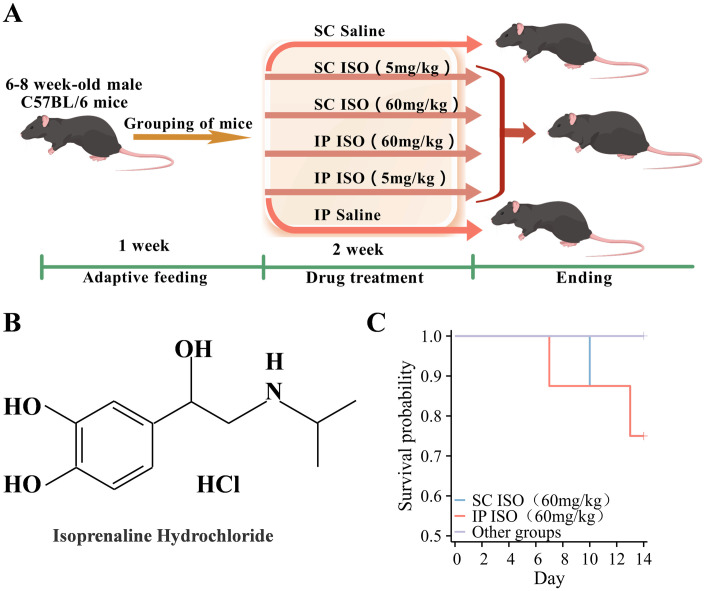
ISO-induced cardiac hypertrophy in mice. **(A)** Experimental design to evaluate ISO-induced HF in C57BL/6J male mice at different doses and routes of administration. **(B)** Chemical structure of ISO hydrochloride. **(C)** The survival rate of mice in each group after two weeks of modeling.

For HF modeling, male C57BL/6J mice aged 6–8 weeks were subjected to subcutaneous (SC) or intraperitoneal (IP) administration of ISO at 5 mg/kg/day (low dose) or 60 mg/kg/day (high dose) for 14 days. Control mice received an equivalent volume of saline using the same administration routes (Fig 1A, 1B). No mortality was observed in either the control or low-dose-treated groups throughout the experimental period. However, in the subcutaneous high-dose group, one animal per timepoint meeting humane endpoint criteria was euthanized on post-modeling days 10, 12, and 13, respectively. Similarly, in the intraperitoneal high-dose group, humane endpoints necessitated euthanasia of one animal on days 7, 8, and 13 post-modeling. These cases were counted as mortality events in survival analysis (Fig 1C).

### ISO-induced cardiac remodeling and altered cardiac function in mice

**Fig 2:**
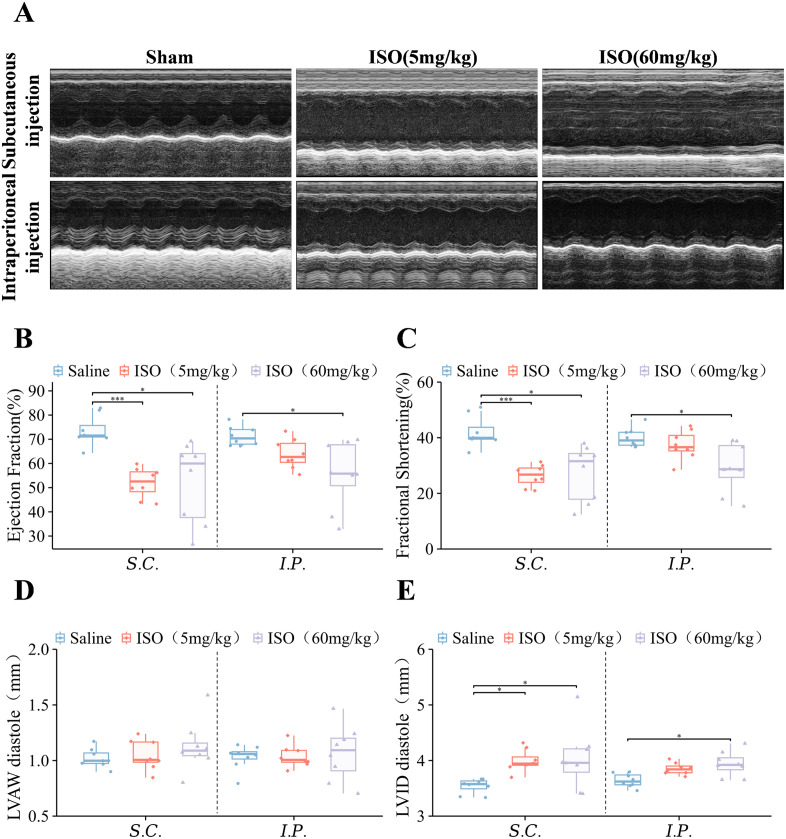
Heart function assessed via echocardiography. For all graphs, symbols represent the number of mice in each cohort (n = 8). **(A)** Representative M-mode echocardiograms. **(B)** % ejection fraction. **(C)** % fractional shortening. **(D)** Diastolic left ventricular anterior wall thickness. **(E)** Diastolic left ventricular internal diameter. Each box-and-line plot represents a line from the minimum to the maximum value, showing all points, with the average value as a line. Statistical significance was determined by two-way ANOVA with post hoc Tukey. *P < 0.05; **P < 0.01 and ***P < 0.001.

We performed echocardiographic analysis to determine whether administration routes and ISO dosages would alter cardiac function. M-mode echocardiographic measurements revealed varying degrees of LVIDd elevation across experimental groups following model establishment, with the SC 60 mg/kg group demonstrating the most pronounced changes (Fig 2A, *P* < 0.001). To systematically evaluate the dose-dependent effects of ISO through different administration routes, we compared cardiac parameters among mice receiving varying ISO doses. Compared with the corresponding saline control group, SC groups exhibited compromised left ventricular systolic function, as evidenced by significant reductions in EF and FS (*P* < 0.001). Notably, the SC 60 mg/kg group exhibited considerable variability in cardiac function. In IP groups, only the IP 60 mg/kg cohort demonstrated statistically significant decreases in EF and FS (Fig 2B, 2C, *P* < 0.05). The LVAWd did not differ significantly among the groups, with a slight increasing tendency observed in the SC ISO groups relative to its control (Fig 2D). Importantly, echocardiographic findings confirmed significant LVIDd elevation in SC-treated mice (*P* < 0.05). Although IP groups showed some LVIDd elevation, statistical significance was only observed in the IP 60 mg/kg cohort (Fig 2E, *P* < 0.05). These data suggest that SC administration induces more pronounced cardiac phenotypic alterations compared to IP treatment when assessed at the 2-week endpoint.

### Differences in ISO dose and route of administration induce significant cardiac hypertrophy and pulmonary index changes

**Fig 3:**
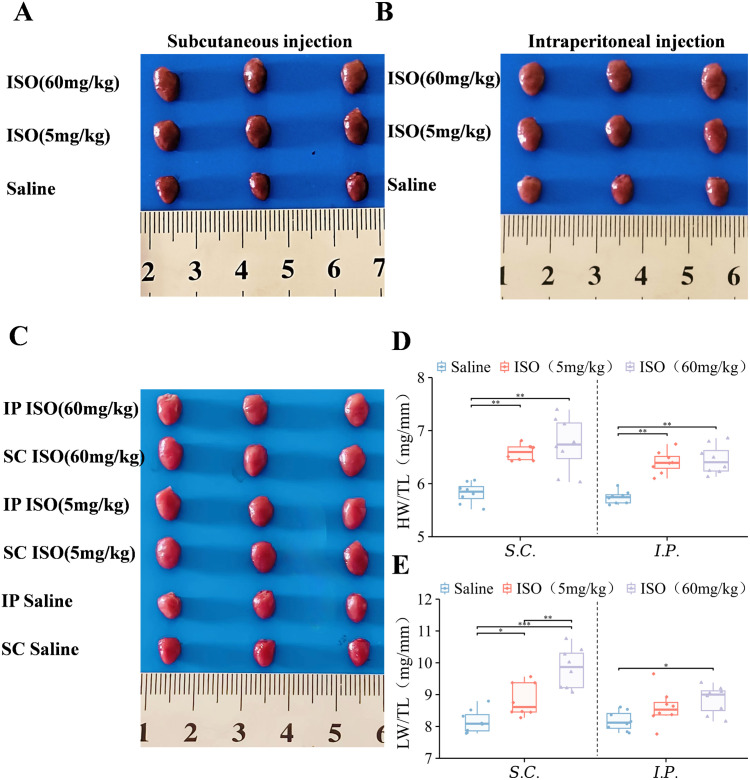
ISO-induced cardiac hypertrophy in mice. For all graphs, symbols represent the number of mice in each cohort (n = 8). **(A)** Representative images of cardiac tissue from subcutaneous injection groups of mice. **(B)** Representative images of cardiac tissue from intraperitoneal injection groups of mice. **(C)** Representative images of cardiac tissue from each group of mice. **(D)** Ratio of heart weight to tibia length in different experimental groups. **(E)** Ratio of lung weight to tibia length in different experimental groups. Each box-and-line plot represents a line from the minimum to the maximum value, showing all points, with the average value as a line. Statistical significance was determined by two-way ANOVA with post hoc Tukey. *P < 0.05; **P < 0.01 and ***P < 0.001.

Subsequently, we measured cardiac and pulmonary weights in mice following ISO administration. Compared with saline-treated controls, experimental groups displayed significantly increased cardiac dimensions, indicating that ISO effectively induced marked cardiac hypertrophy regardless of dosage (Fig 3A, 3B, 3C). Further evaluation of administration routes and ISO dosages revealed consistent findings: the SC 5 mg/kg group demonstrated more pronounced and stable differences compared to other experimental cohorts (Fig 3D).

Notably, the pulmonary indices in all experimental groups were significantly increased compared to the control group (*P* < 0.05), with statistically significant differences observed between the high-dose subcutaneous and low-dose subcutaneous groups (*P* < 0.01). These data demonstrate the greater pulmonary impacts from high-dose ISO and more pronounced effects via SC administration (Fig 3E). Collectively, our results demonstrate that ISO exacerbates cardiopulmonary weight alterations regardless of dosage magnitude or delivery method.

### ISO-induced myocardial fibrosis in mice

**Fig 4:**
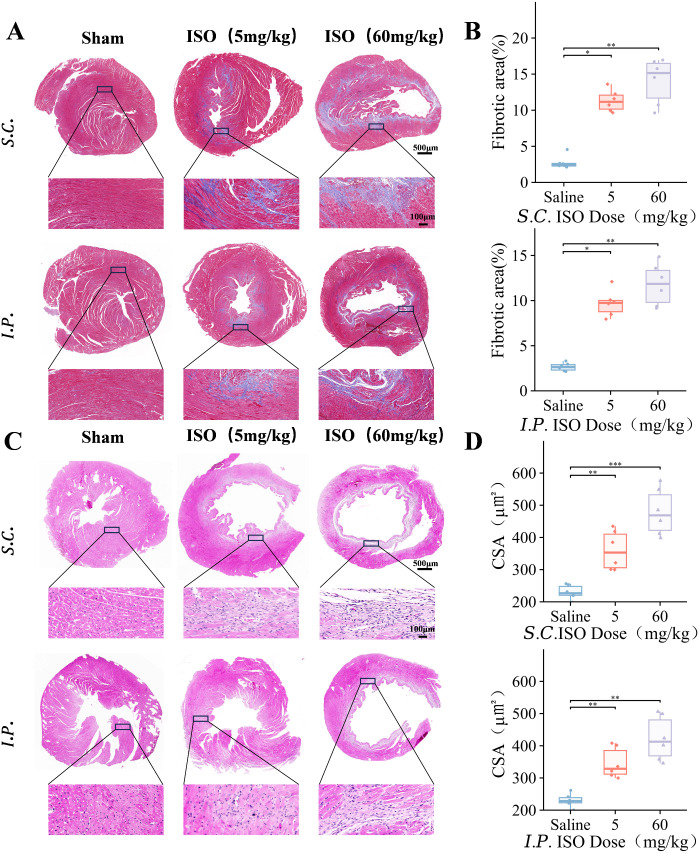
Myocardial fibrosis and cardiac hypertrophy induced by different doses and routes of administration of ISO in C57BL/6J mice. For all graphs, symbols represent the number of mice in each cohort (n = 6). **(A)** Representative images of Masson's trichrome stained. **(B)** Area of fibrosis in different groups of mice. **(C)** Representative HE staining images. **(D)** Cross-sectional area of cardiomyocytes (CSA) in different groups of mice. Each box-and-line plot represents a line from the minimum to the maximum value, showing all points, with the average value as a line. Statistical significance was determined by two-way ANOVA with post hoc Tukey. *P < 0.05; **P < 0.01 and ***P < 0.001.

To systematically evaluate the effect of ISO on cardiac remodeling in mouse HF models, myocardial tissue specimens from experimental cohorts underwent comprehensive histopathological evaluation. Masson’s trichrome staining demonstrated pronounced collagen deposition (blue staining) in left ventricular sections of experimental groups, with collagen fibers exhibiting linear arrangements and focal coalescence patterns (Fig 4A). Quantitative analysis revealed significantly exacerbated myocardial fibrosis in high-dose ISO cohorts (*P* < 0.01), while the IP 5 mg/kg group showed relatively slighter fibrotic alterations (Fig 4B). Corresponding H&E staining micrographs indicated that myocardial architecture in ISO-treated groups was extensively supplanted by fibrotic scar tissue, accompanied by extensive cardiomyocyte necrosis (Fig 4C). Persistent cardiomyocytes exhibited compensatory cellular hypertrophy and proliferative changes, characterized by nuclear enlargement and cytoplasmic expansion (Fig 4D).

### ISO promotes expression of hypertrophic and fibrotic gene markers

**Fig 5:**
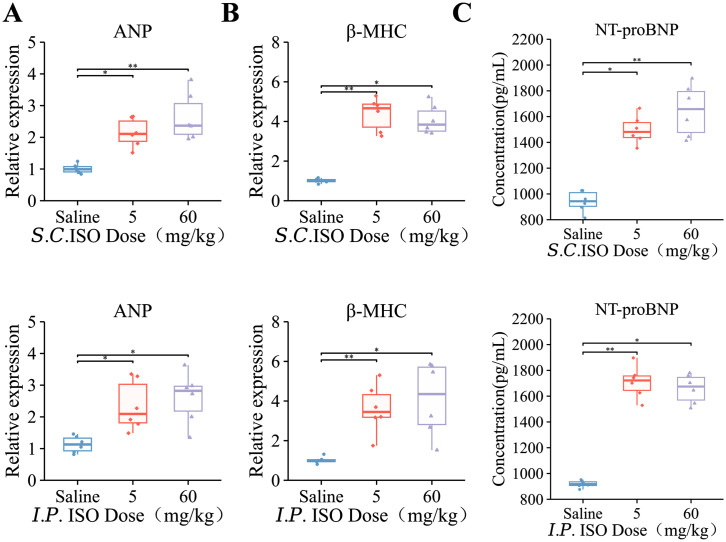
ISO elevates levels of myocardial fibrosis and cardiac hypertrophy makers in C57BL/6J mice. For all graphs, symbols represent the number of mice in each cohort (n = 6). **(A)** The mRNA levels of ANP in the heart tissues based on real-time PCR. GAPDH mRNA was used as loading control. **(B)** The mRNA levels of β-MHC in the heart tissues based on real-time PCR. GAPDH mRNA was used as loading control. **(C)** NT-proBNP level in the serum based on ELISA. Each box-and-line plot represents a line from the minimum to the maximum value, showing all points, with the average value as a line. Statistical significance was determined by two-way ANOVA with post hoc Tukey. *P < 0.05; **P < 0.01 and ***P < 0.001.

Next, we investigated whether ISO dosage and delivery routes differently induced cellular hallmarks of myocardial hypertrophy and fibrosis in mice. Our data revealed no significant impact of administration routes alone on cardiac hypertrophic marker expression. However, compared with the control group, atrial natriuretic peptide (ANP) showed significant elevation across all experimental cohorts (Fig 5A, *P* < 0.05). When examining β-myosin heavy chain (β-MHC), a more pronounced increasing trend was detected in the low-dose ISO groups (*P* < 0.01), and the overall trend of elevation was more stable for both hypertrophic markers (Fig 5B).

Cardiac functional assessment through serum N-terminal pro-B-type natriuretic peptide (NT-proBNP) quantification revealed significant intergroup differences, with observed effects mediated specifically by ISO dosage rather than delivery routes (Fig 5C). These findings collectively confirm previously reported hypertrophic and fibrotic effects of low-dose ISO administration, while demonstrating route-independent biological responses. The subcutaneous delivery method combined with a low-dose ISO regimen appears optimal for establishing stable pathological myocardial remodeling.

## Discussion

In the present study, we report a comparison of two doses and two delivery methods to induce HF in C57BL/6J mice. Using pathophysiological and molecular biological analyses, we identified key limitations of high-dose ISO administration (60 mg/kg/day): High-dose ISO treatment was accompanied by substantial mortality risk, while demonstrating significant inter-individual variability in pathological manifestations. In contrast, the low-dose regimen (5 mg/kg/day) achieved consistent HF induction, showing superior stability in key parameters including left ventricular ejection fraction and collagen deposition. Comparative analysis of administration routes further identified subcutaneous delivery as optimal, yielding significantly better preservation of cardiac structure-function relationships than intraperitoneal injection.

ISO is a non-selective β-adrenergic agonist which is widely utilized in cardiovascular research for establishing HF models [11]. The ISO-induced HF model serves as a critical platform for investigating cardiac pathophysiological mechanisms, drug efficacy, and novel therapeutic interventions [12,13]. Studies demonstrate that ISO triggers HF pathogenesis through β-adrenergic receptor activation, leading to metabolic dysregulation, aberrant calcium handling, and myocardial remodeling in cardiomyocytes [14]. However, variability in reported dosages and administration routes has hindered the identification of an optimal ISO dosing and delivery protocol for generating consistent and reliable injury models in mice. For instance, Wang *et al.* used a decreasing-concentration regimen via SC injection, whereas Dong *et al.* used a fixed-dose ISO regimen (5 mg/kg/day) subcutaneously for 14 consecutive days [15,16]. Similarly, Wu *et al.* induced the model by IP injection of a constant concentration of ISO for 28 days [17], while some researchers have used osmotic minipumps (60 mg/kg/day) for continuous ISO delivery to induce chronic HF [18]. Due to these divergent approaches, there is currently no clear or standardized experimental protocol for ISO-induced HF modeling, and objective evaluation criteria remain lacking.

Studies have demonstrated low and high doses of ISO engage distinct mechanistic pathways. Low-dose ISO predominantly activates myocardial hypertrophy signaling pathways (e.g., the MAPK pathway), enhancing myocardial contractility and thereby improving cardiac pumping capacity [19]. In contrast, high-dose ISO induces excessive β1-adrenergic receptor stimulation, resulting in cardiomyocyte calcium overload, mitochondrial dysfunction, and reactive oxygen species (ROS) overproduction, which directly triggers necrotic/apoptotic cell death rather than compensatory hypertrophy [6,20,21]. In this study, we administered ISO at two doses (5 or 60 mg/kg/day) over two weeks to assess whether SC and IP delivery confer comparable cardiac injury risks in male C57BL/6J mice. Interestingly, the degree of cardiac hypertrophy showed minimal divergence between low- and high-dose groups. This apparent plateau in hypertrophic response suggests a ceiling effect, beyond which further β-adrenergic stimulation fails to amplify the effect. Several non-exclusive mechanisms could explain this non-linear dose-response relationship, including potential β-receptor desensitization, feedback inhibition of pro-hypertrophic signaling cascades, or a shift toward necrotic remodeling at higher doses that may counteract transcriptional activation of hypertrophy genes. This observation may reflect a pathological compensatory saturation effect, suggesting that even low-dose ISO can maximally activate pathological remodeling pathways in this model. Our findings can be contextualized among established protocols. For instance, some protocols employ a sustained moderate dose (20 mg/kg/day for 25 days) to achieve optimal HF induction, while others utilize an intensive short-term regimen (100 mg/kg/day for 7 days) followed by a recovery period to establish a stable heart failure with reduced ejection fraction (HFrEF) phenotype [22,23]. Our model tested two distinct strategies: a low-dose (5 mg/kg/day) protocol favoring survivability and gradual dysfunction, and a high-dose (60 mg/kg/day) approach that balances phenotypic severity with mortality. Notably, the 14-day SC administration of 5 mg/kg/day ISO consistently induced a robust and stable HF phenotype. Although the high-dose regimen produced more severe injury in some measures, it also introduced greater variability and higher mortality without significantly augmenting hypertrophic response. Thus, for studies focused on hypertrophy and chronic remodeling, the lower dose offers a preferable balance between phenotypic robustness and survival.

Intraperitoneal ISO administration enables rapid drug absorption via peritoneal vasculature, effectively mimicking catecholamine-induced cardiac remodeling. However, it carries risks of local complications (pain, infection) and systemic adverse effects (peritonitis, organ damage), particularly with improper technique [24,25]. Subcutaneous injection is simple to operate and can realize the slow release of the drug, avoiding the rapid metabolism of the drug in the body, and can maintain a more stable blood concentration. However, subcutaneous injection also has some limitations. First, the absorption rate of subcutaneous tissue is relatively slow, which may lead to a prolonged onset of drug effects. Second, for some drugs, subcutaneous injection may lead to local reactions, such as redness, swelling, pain, or allergic reactions, which may affect the normal physiological state of mice [26]. Cardiac functional assessments via echocardiography revealed comparable alterations in both SC and IP ISO delivery groups. This divergence may be attributed to reduced first-pass elimination in SC delivery, which enhances drug bioavailability and amplifies pharmacological effects, whereas IP administration likely facilitates accelerated hepatic metabolism and reduces drug utilization [27]. Consistent with these functional observations, histopathologic analyses of myocardial tissue also confirmed the variability in routes of administration. SC-administered mice displayed elevated myocardial fibrosis severity relative to IP groups at matched doses, as quantified by Masson's trichrome staining. Similarly, HE staining revealed exacerbated cardiomyocyte necrosis and expanded scar tissue areas in SC-treated cohorts. These collective findings underscore the critical influence of administration routes on ISO-induced HF severity, with SC delivery emerging as the preferential modality for achieving robust and reproducible pathological phenotypes.

While the present findings elucidate the complexities of low-dose ISO administration and route-dependent effects, several limitations warrant consideration. First, statistical power was constrained by cohort size and data variability, necessitating cautious interpretation of negative results. Second, although both groups exhibited significant myocardial dysfunction, observed divergences in select echocardiographic parameters (e.g., diastolic function) and molecular markers lacked sufficient evidence to conclusively establish route-specific causality. Finally, although mouse models are genetically manipulable, future validation in rat models—which allow for repeated hemodynamic measurements—will enhance their translational relevance. This remains a focus of current research. Future studies integrating longitudinal assessments and expanded sample sizes are required to validate these preliminary observations.

## Materials and methods

### Ethic statement

This research conformed to the Guide for the Care and Use of Laboratory Animals published by the US National Institute of Health (NIH Publication, 8th Edition, 2011). All experimental protocols were approved by the Ethics Committee of Bengbu Medical University (protocol No. 2025–560). Research staff had received specialized training in animal care and handling, including animal ethics (3R principles, local regulations) and technical skills (restraint, injection, euthanasia). The mice were fed in a specific pathogen-free environment (12/12h light/darkness, 45–60% relative humidity, and 24–26°C room temperature) with free access to food and water. The overall health status was checked by trained professionals. All efforts were made to minimize animal suffering.

During the experiment, animals’ wellbeing was followed every 12 hours for signs of distress and endpoints. The following predefined humane endpoints were employed for euthanasia: moribund state (inability to maintain upright posture with or without labored breathing/cyanosis), or fulfillment of ≥4 criteria from: > 20% weight loss, anorexia, sustained hunched posture (>12h), prostration, motility impairment, dyspnea, ruffled fur, or dehydration. Mice were euthanized by cervical dislocation after induction of anesthesia (inhalation of isoflurane) upon meeting humane endpoints. Euthanized mice were considered as non-survivors.

### Establishment of HF model

For HF modeling, male C57BL/6J mice aged 6–8 weeks were subjected to subcutaneous (SC) or intraperitoneal (IP) administration of ISO (cat. # HY-B0468, MCE) at two literature-established doses (5 and 60 mg/kg/day for 14 days). Briefly, the animals were randomly divided into six cohorts (n = 12 for each group): (1) SC saline control, (2) IP saline control, (3) SC 5 mg/kg ISO, (4) IP 5 mg/kg ISO, (5) SC 60 mg/kg ISO, and (6) IP 60 mg/kg ISO, with daily administration for 14 days. ISO was dissolved in sterile saline (0.9% NaCl) immediately before each injection. At the end of modeling, mice were anaesthetized to observe the cardiac function using echocardiography, and then sacrificed with hearts and serum harvested for subsequent experiments.

Three animals each from SC 60 mg/kg ISO group and IP 60 mg/kg ISO group were euthanized upon reaching humane endpoints before conclusion of the study. Their data were included solely in mortality statistics and excluded from all other analyses. No animal died before meeting criteria for euthanasia.

### Echocardiographic detection

Cardiac ultrasound examinations were performed post-modeling following standardized protocols. Mice were anesthetized with 2% isoflurane via nasal inhalation and positioned in a supine position on a temperature-controlled platform. M-mode echocardiograms were acquired using the MX-400 high-frequency transducer (30 MHz) positioned at the parasternal short-axis view at the papillary muscle level. Cardiac architecture and functional parameters were quantified using a high-resolution small-animal ultrasound system (VisualSonics Vevo 2100) with the following measurements: left ventricular anterior wall thickness at end-diastole (LVAW; d) and left ventricular internal dimension at end-diastole (LVID; d). Derived functional indices, including ejection fraction (EF) and fractional shortening (FS), were calculated using established formulas.

### Enzyme-linked immunosorbent assay to measure N-terminal brain natriuretic

Peptide precursor serum NT-proBNP levels were analyzed using a commercially available ELISA kit (cat. # JL11641, Shanghai Jianglai Biotechnology) according to the manufacturer's recommended protocols.

### Histopathological examination of heart tissue

Heart tissues were routinely collected, fixed, processed and sectioned. Hematoxylin and eosin (HE) staining was performed on 4-μm paraffin sections. After deparaffinization and rehydration, sections were stained with Mayer's hematoxylin, differentiated in 0.5% acid alcohol, counterstained with eosin Y, and mounted with resin. Each group consisted of 6 samples, and 3 regions were randomly selected from each sample, in which the cross-sectional area of cardiomyocytes was counted by ImageJ (v1.53). For fibrosis detection, Masson's trichrome staining was implemented: sections were incubated sequentially in Bouin's fixative (60°C, 1 h), Weigert's iron hematoxylin (10 min), Biebrich scarlet-acid fuchsin (5 min), and aniline blue (5 min). Collagen deposition (blue) and cardiomyocytes (red) were visualized under polarized light. Images were acquired at a magnification of 400 × . The fraction of myocardial volume occupied by fibrillar collagen was calculated as the percent total surface area occupied by the interstitial fibrosis.

### Expression levels of ANP and β-MHC

Total RNA was isolated from cardiac tissues using TRIzol reagent (cat. # G3013, Servicebio). Complementary DNA (cDNA) was synthesized using the PrimeScript™ RT Reagent Kit (cat. # R333-01, Vazyme). Quantitative reverse transcription-polymerase chain reaction (qRT-PCR) was performed using SYBR Green PCR Master Mix (cat. # 11201ES03, Yeasen). The sequences of primers are as follows: ANP, 5’-AGGTCGGTGTGAACGGATTTG-3’ and 5’-TGTAGACCATGTAGTTGAGGTCA-3’; β-MHC, 5’-TCTTCCTCGTCTTGGCCTTT-3’, and 5’-CCAGGTGGTCTAGCAGGTTC-3’; GAPDH, 5’-AGGTCGGTGTGAACGGATTTG-3’ and 5’-TGTAGACCATGTAGTTGAGGTCA-3’. Relative mRNA expression levels were normalized to GAPDH and calculated via the 2^(-ΔΔCt) method.

### Statistical analysis

All experimental data are expressed as mean ± standard deviation (SD). Statistical analyses were performed using GraphPad Prism 8.0.1. Intergroup differences were evaluated by two-way ANOVA with post hoc tukey. A probability value of *P* < 0.05 was considered statistically significant for all comparisons.

## Conclusions

In summary, this study demonstrates that sustained β-adrenergic receptor stimulation via either SC or IP ISO administration for 2 weeks induces comparable cardiac pathological phenotypes in C57BL/6J mice. However, SC delivery of ISO at 5 mg/kg/day emerged as the most stable and reliable protocol for modeling chronic HF. A critical direction for future research lies in addressing the multifaceted pathogenesis of HF, a complex clinical syndrome that entails intricate crosstalk across multiple systems and cellular compartments. Current preclinical models generated through varying combinations of drug dosages and administration routes engage distinct pathophysiological mechanisms, underscoring the need for methodological refinement. Emerging CHF research must prioritize precision-driven and individualized paradigms to faithfully recapitulate human disease mechanisms. Such advancements will provide mechanistically grounded rationales for early diagnosis and personalized therapeutics, ultimately bridging the translational gap between experimental models and clinical management.

## Supporting information

S1 File (ZIP)
