# Supplementary figures and images for: Heart failure induced by isoproterenol: A comparison of two doses and two delivery methods in C57BL/6J mice
Source: PLoS One. 2025 Nov 3;20(11):e0334880. doi: 10.1371/journal.pone.0334880 (PMC12582447; doi:10.1371/journal.pone.0334880)

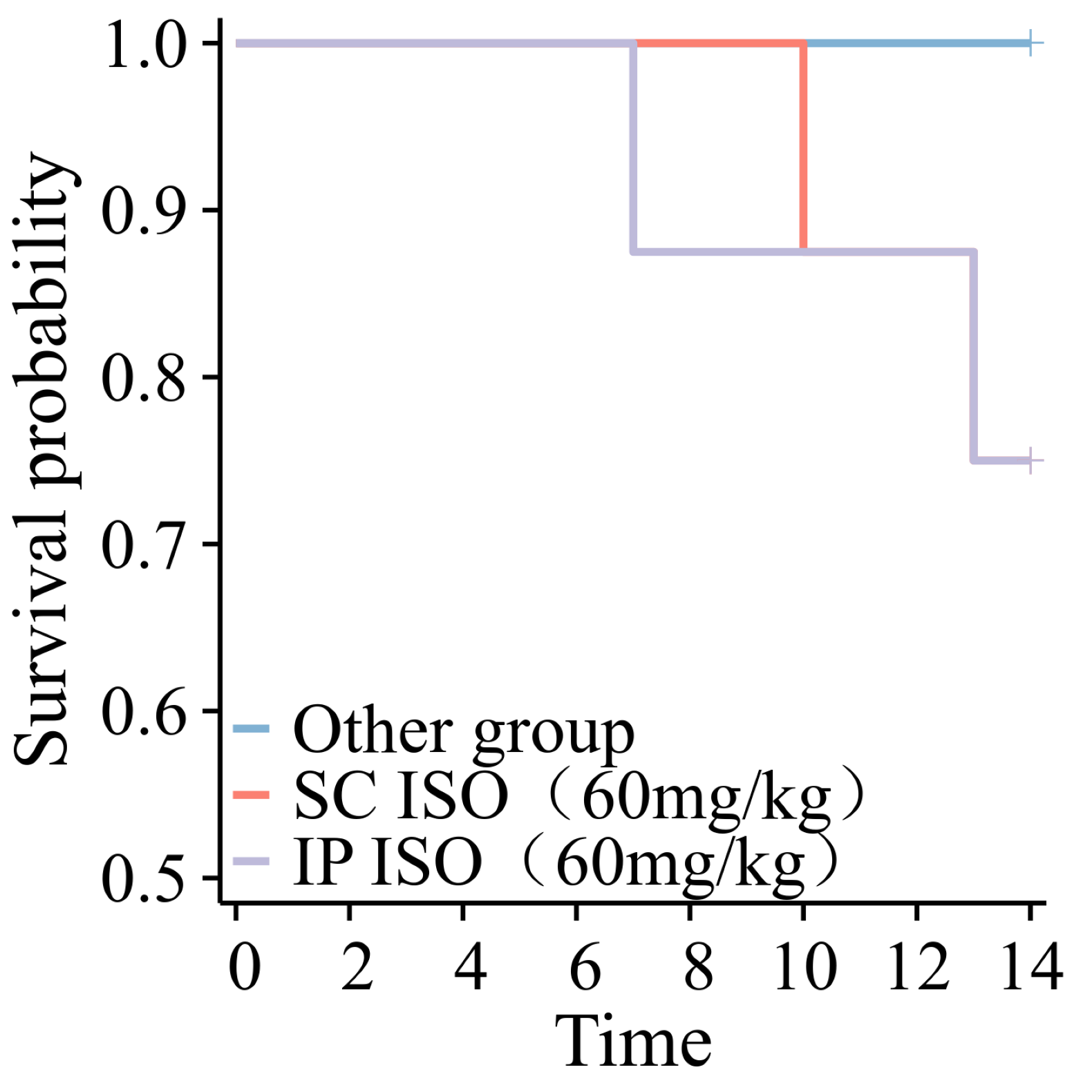

Supplement: S1 File — (ZIP) [file pone.0334880.s001.zip › Supporting information files20251008/Data set for Figure 1/Fig1-C/Survival probability.tif]

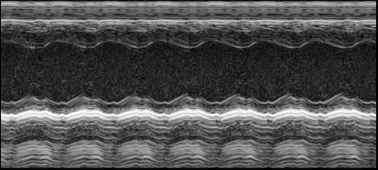

Supplement: S1 File — (ZIP) [file pone.0334880.s001.zip › Supporting information files20251008/Data set for Figure 2/Fig2-A/Representative images of M-mode in each group/IP ISO 5mg-kg.tif]

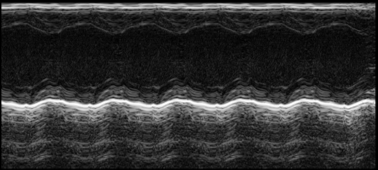

Supplement: S1 File — (ZIP) [file pone.0334880.s001.zip › Supporting information files20251008/Data set for Figure 2/Fig2-A/Representative images of M-mode in each group/IP ISO 60mg-kg.tif]

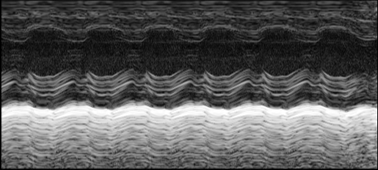

Supplement: S1 File — (ZIP) [file pone.0334880.s001.zip › Supporting information files20251008/Data set for Figure 2/Fig2-A/Representative images of M-mode in each group/IP Sham.tif]

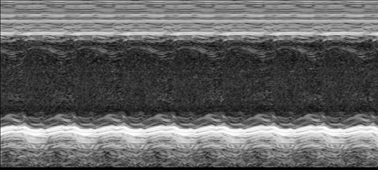

Supplement: S1 File — (ZIP) [file pone.0334880.s001.zip › Supporting information files20251008/Data set for Figure 2/Fig2-A/Representative images of M-mode in each group/SC ISO 5mg-kg.tif]

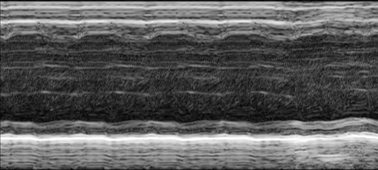

Supplement: S1 File — (ZIP) [file pone.0334880.s001.zip › Supporting information files20251008/Data set for Figure 2/Fig2-A/Representative images of M-mode in each group/SC ISO 60mg-kg.tif]

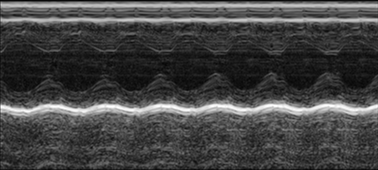

Supplement: S1 File — (ZIP) [file pone.0334880.s001.zip › Supporting information files20251008/Data set for Figure 2/Fig2-A/Representative images of M-mode in each group/SC Sham.tif]

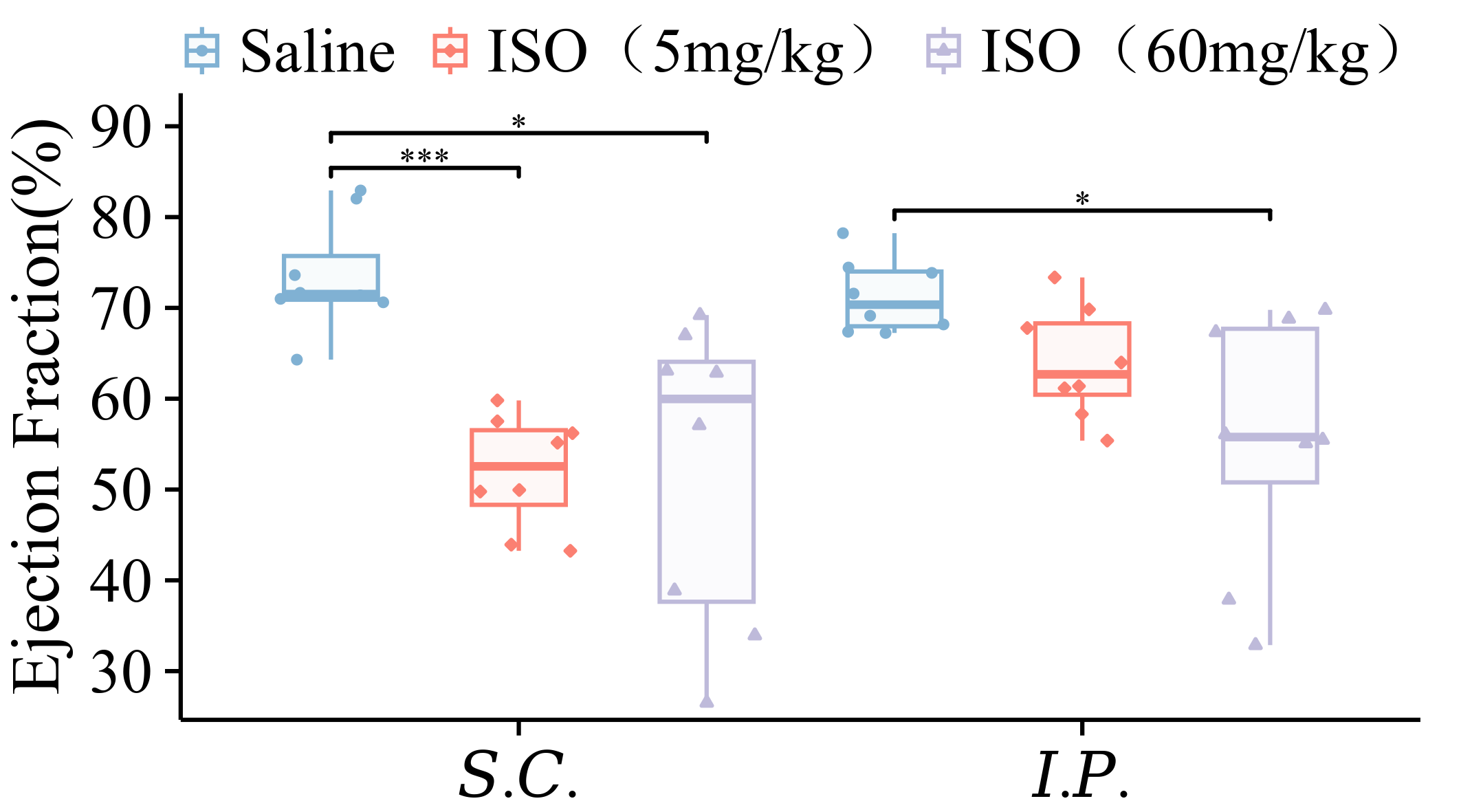

Supplement: S1 File — (ZIP) [file pone.0334880.s001.zip › Supporting information files20251008/Data set for Figure 2/Fig2-B-E/Box statistical charts of EF values for each group.tiff]

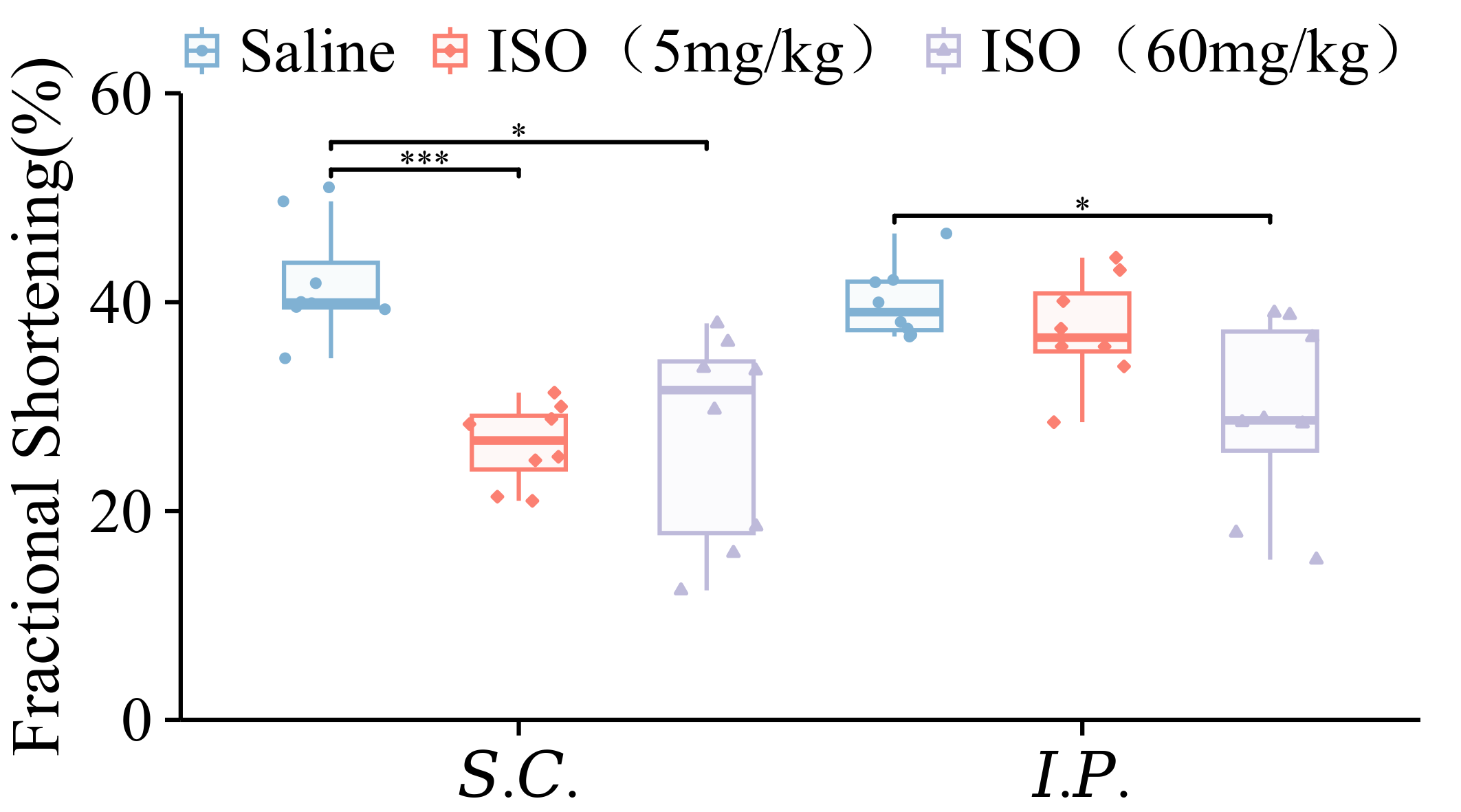

Supplement: S1 File — (ZIP) [file pone.0334880.s001.zip › Supporting information files20251008/Data set for Figure 2/Fig2-B-E/Box statistical charts of FS values for each group.tiff]

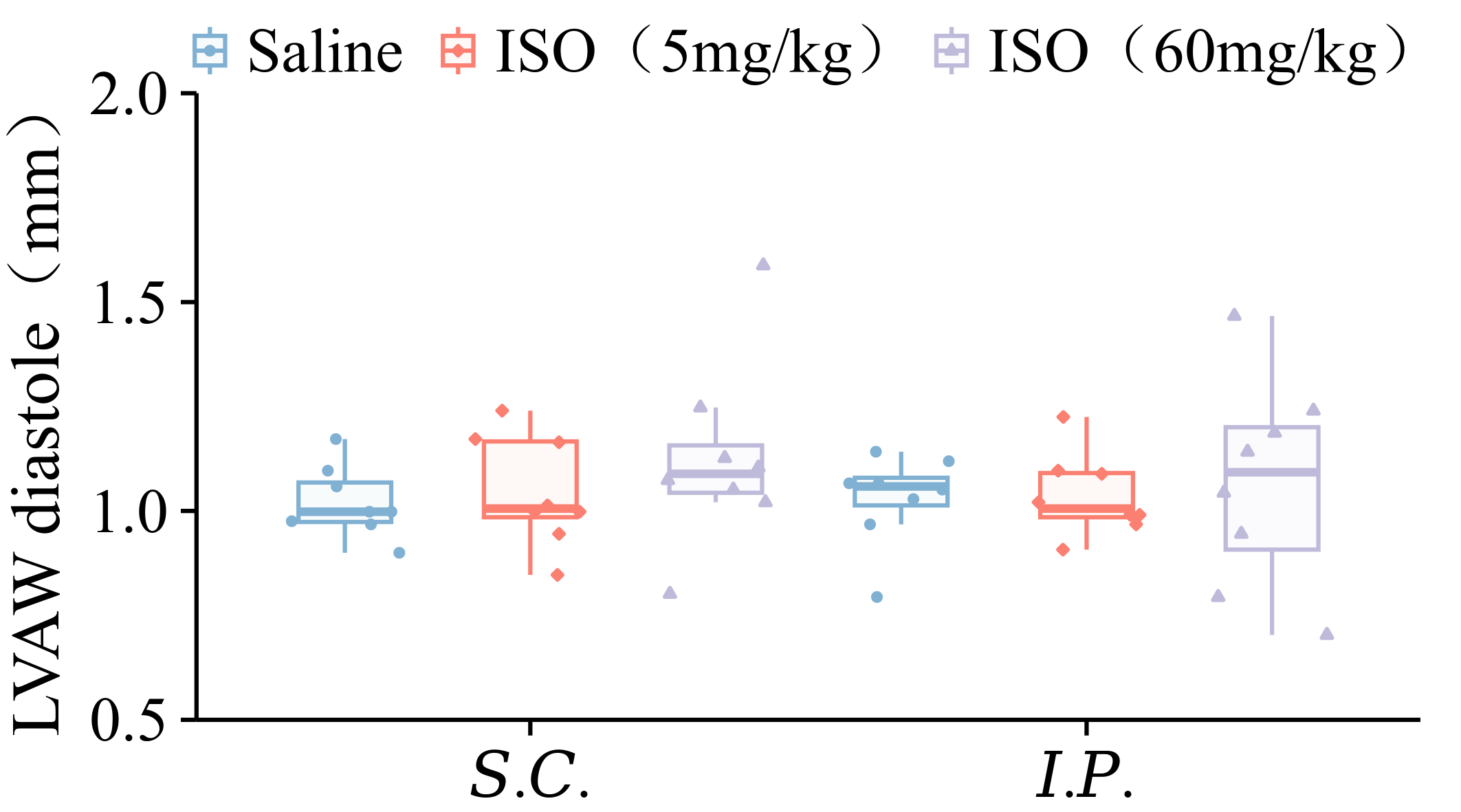

Supplement: S1 File — (ZIP) [file pone.0334880.s001.zip › Supporting information files20251008/Data set for Figure 2/Fig2-B-E/Box statistical charts of LVAWd values for each group.tiff]

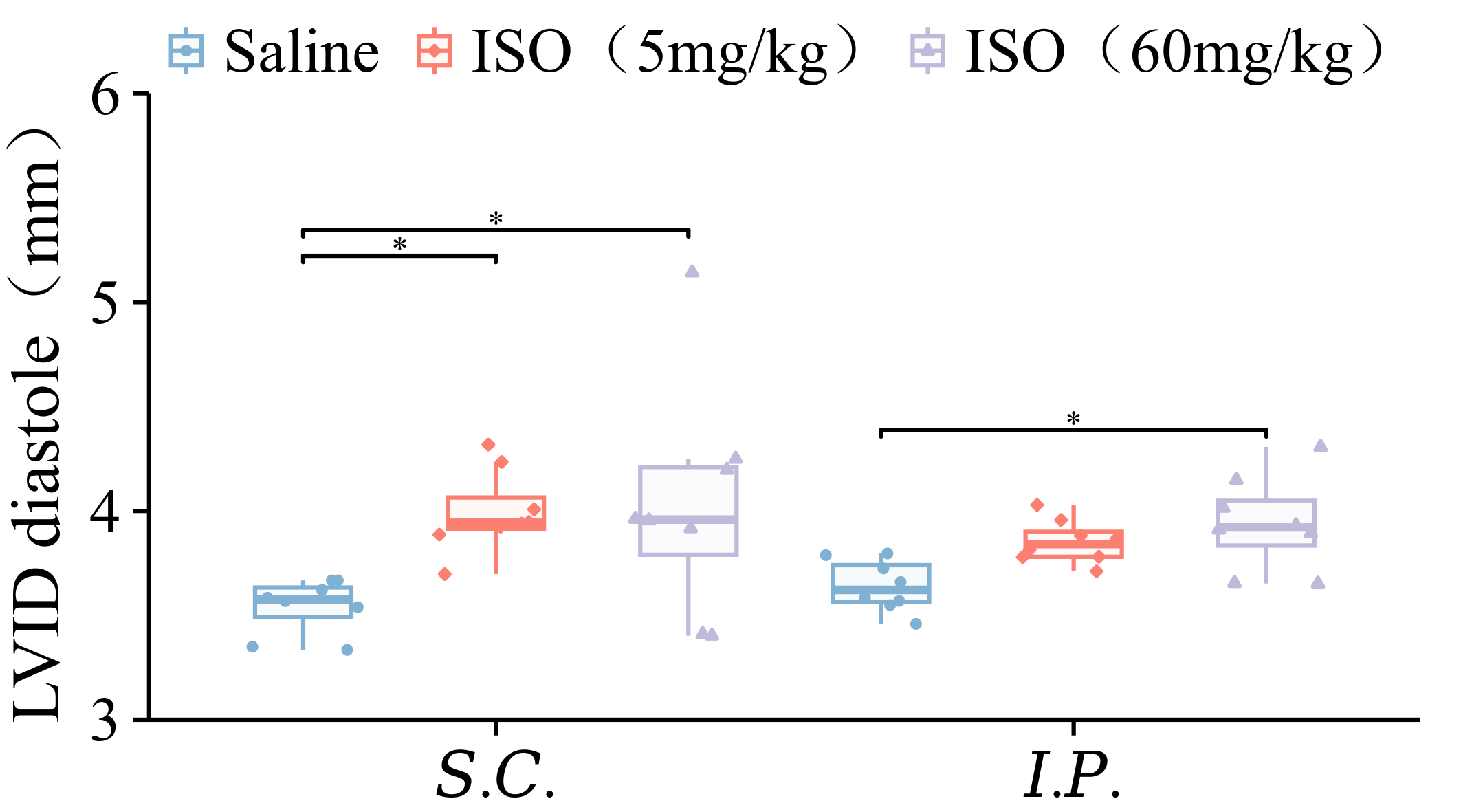

Supplement: S1 File — (ZIP) [file pone.0334880.s001.zip › Supporting information files20251008/Data set for Figure 2/Fig2-B-E/Box statistical charts of LVID values for each group.tiff]

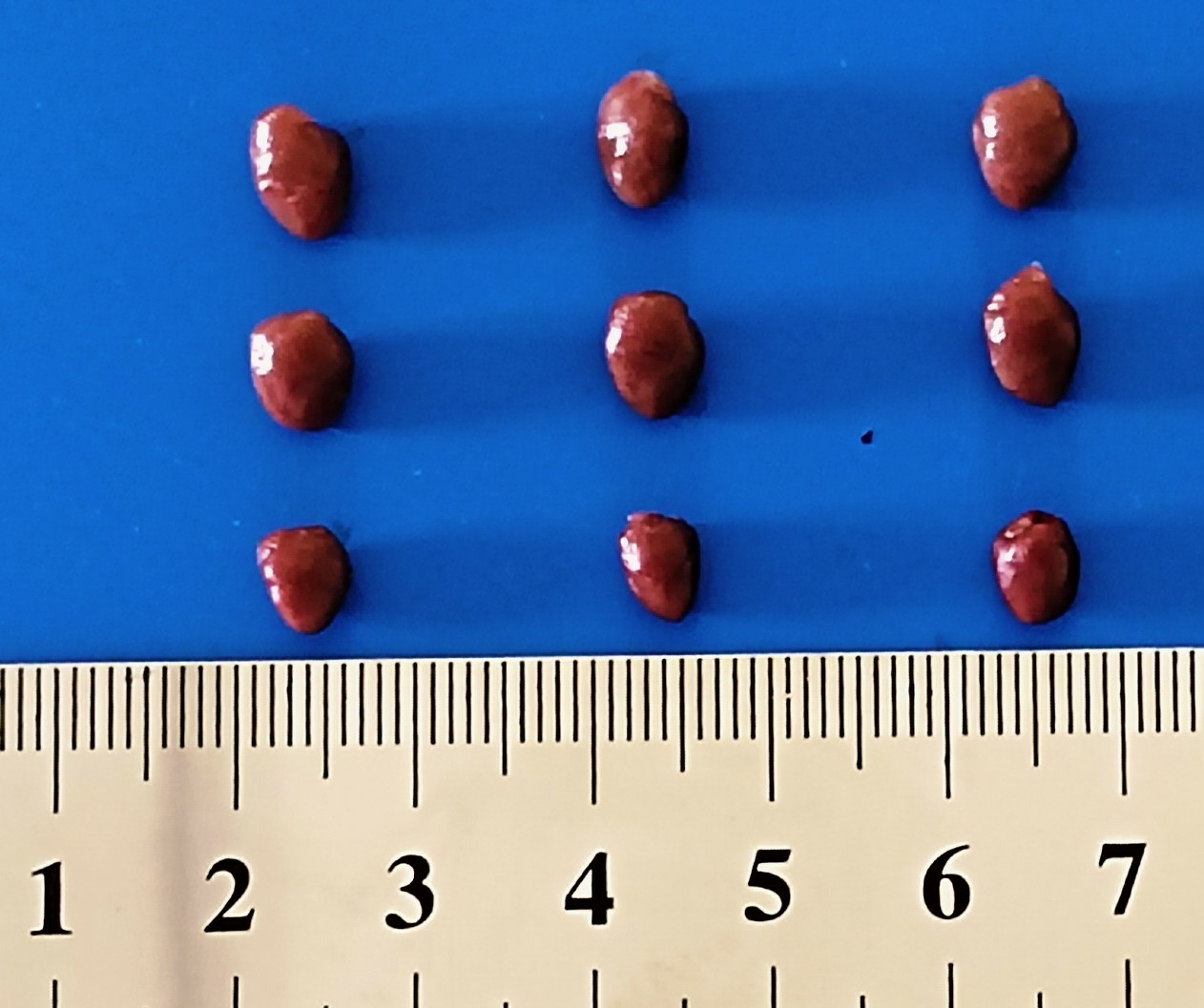

Supplement: S1 File — (ZIP) [file pone.0334880.s001.zip › Supporting information files20251008/Data set for Figure 3/Fig3A-C Representative cardiac images of each group/Fig3A Representative cardiac images of SC group.tif]

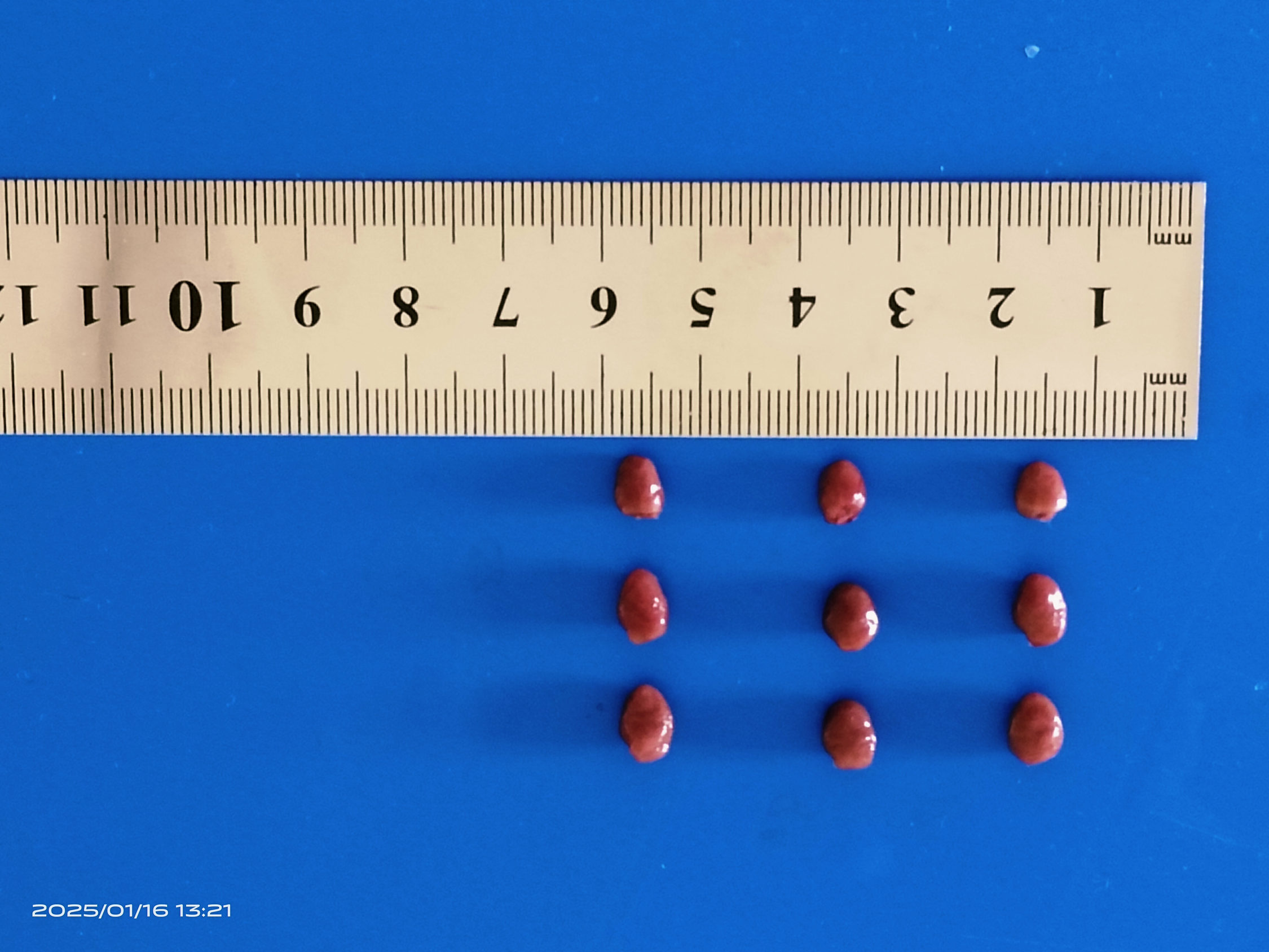

Supplement: S1 File — (ZIP) [file pone.0334880.s001.zip › Supporting information files20251008/Data set for Figure 3/Fig3A-C Representative cardiac images of each group/Fig3B Representative cardiac images of IP group.tif]

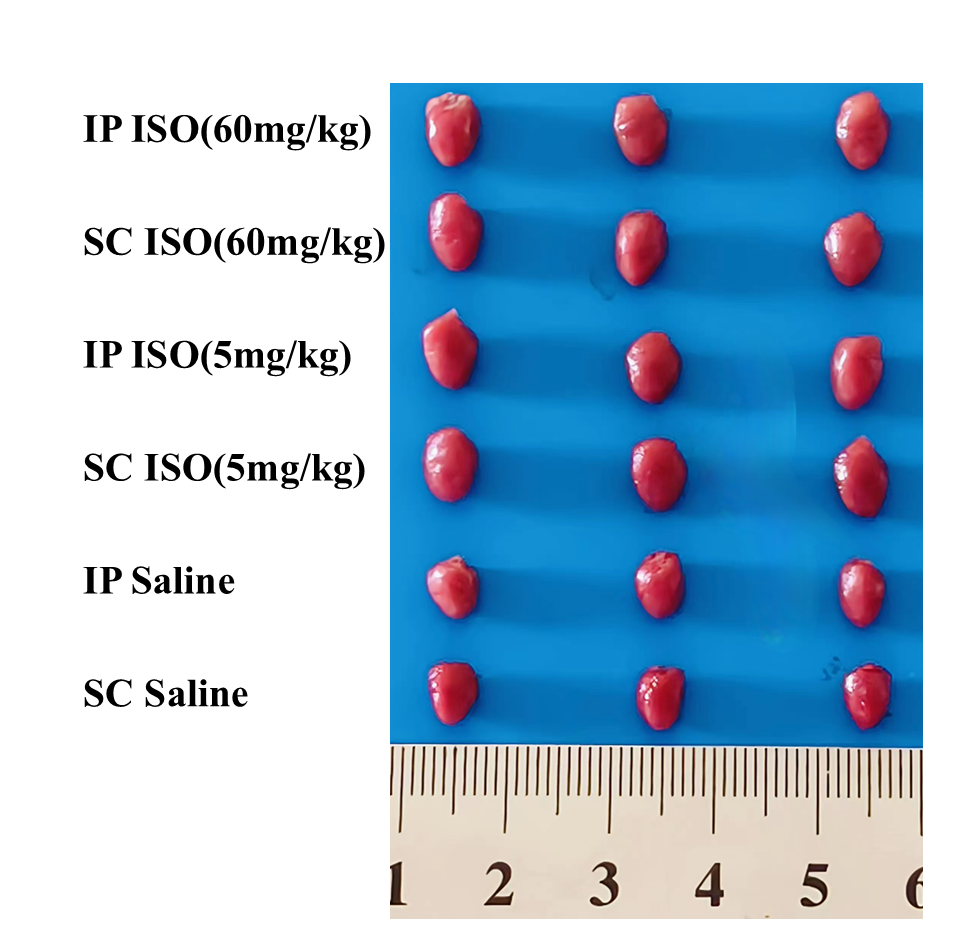

Supplement: S1 File — (ZIP) [file pone.0334880.s001.zip › Supporting information files20251008/Data set for Figure 3/Fig3A-C Representative cardiac images of each group/Fig3C Representative cardiac images of each group.tif]

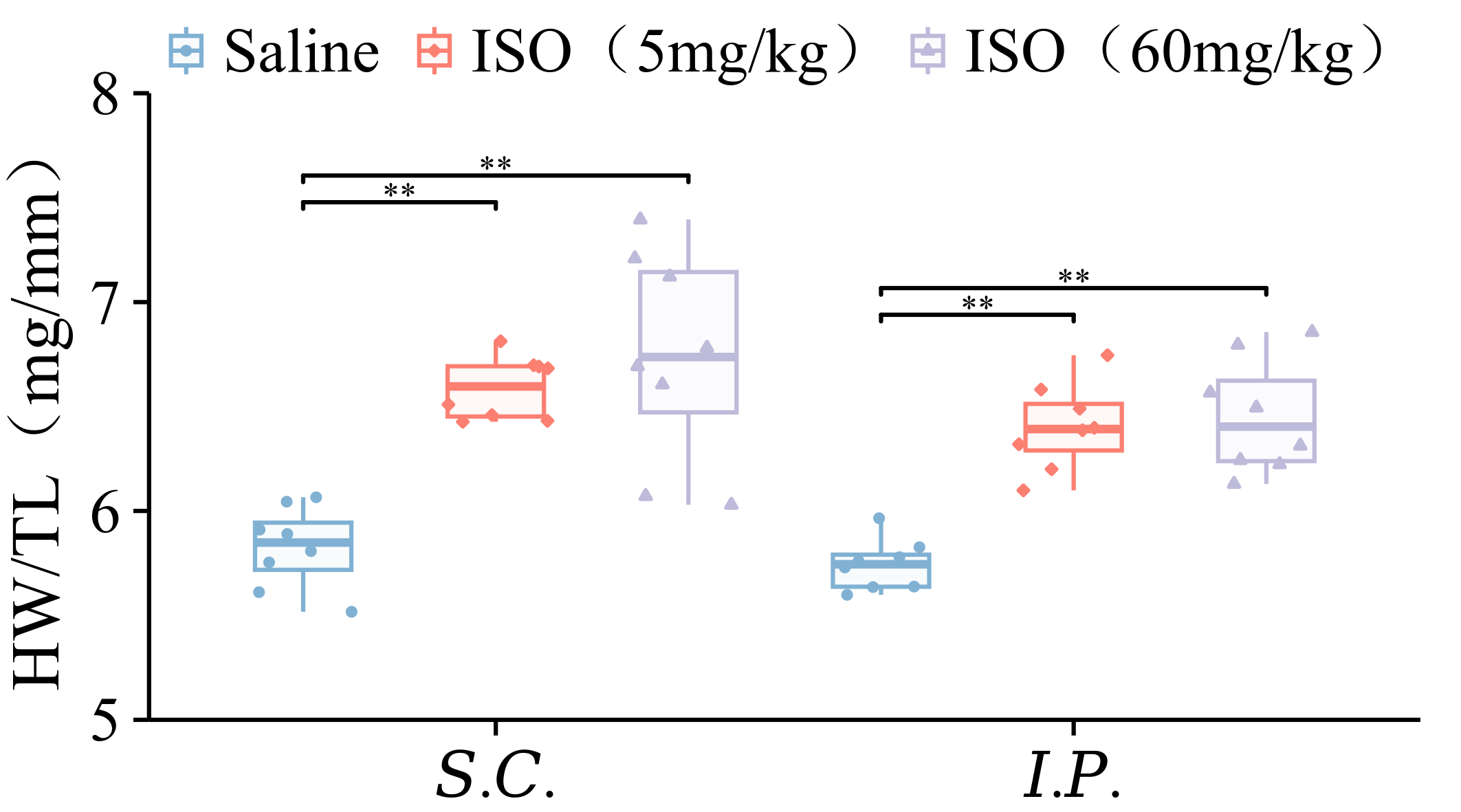

Supplement: S1 File — (ZIP) [file pone.0334880.s001.zip › Supporting information files20251008/Data set for Figure 3/Fig3D-E/Box-type statistical charts of HWTL in each group.tif]

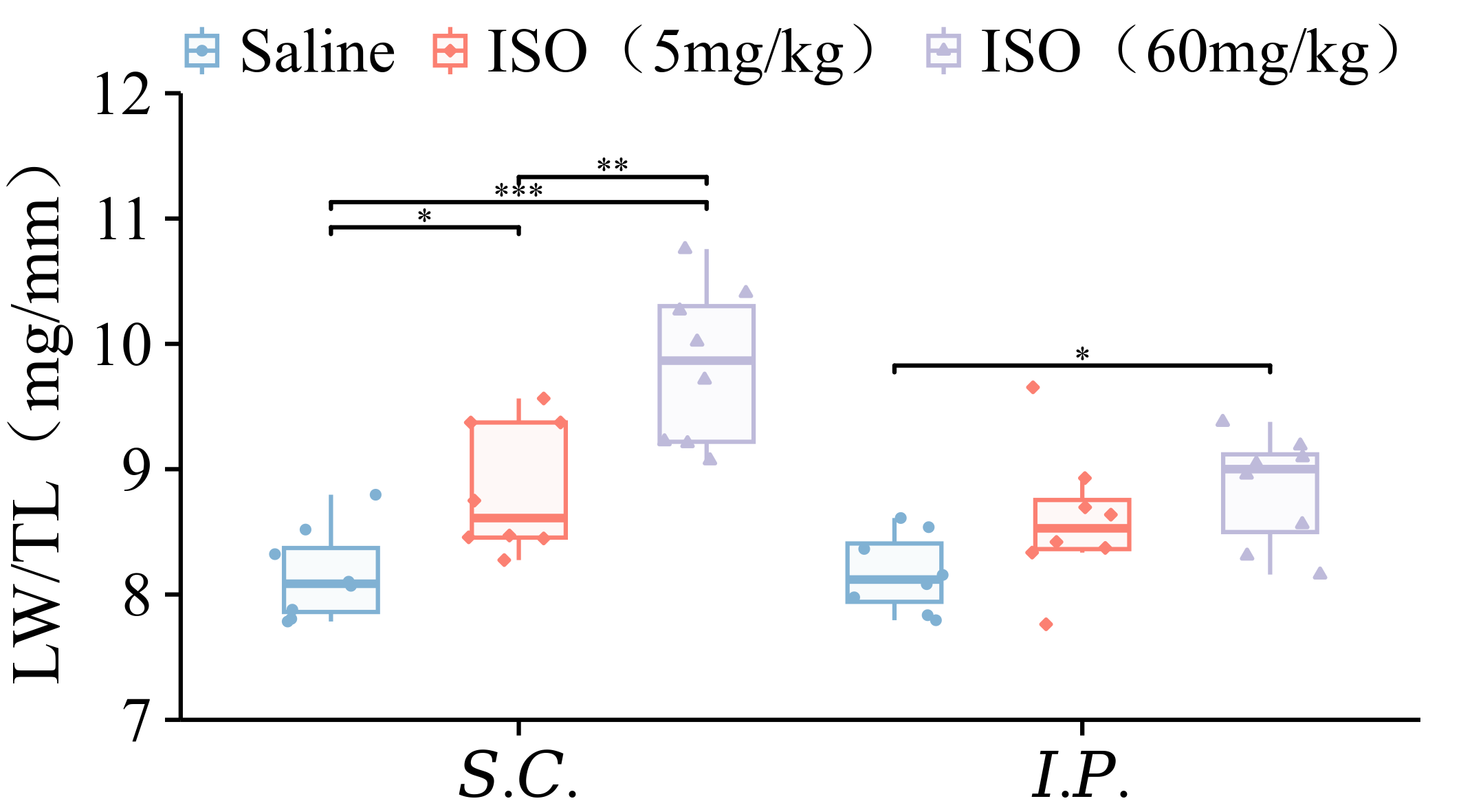

Supplement: S1 File — (ZIP) [file pone.0334880.s001.zip › Supporting information files20251008/Data set for Figure 3/Fig3D-E/Box-type statistical charts of LWTL in each group.tif]

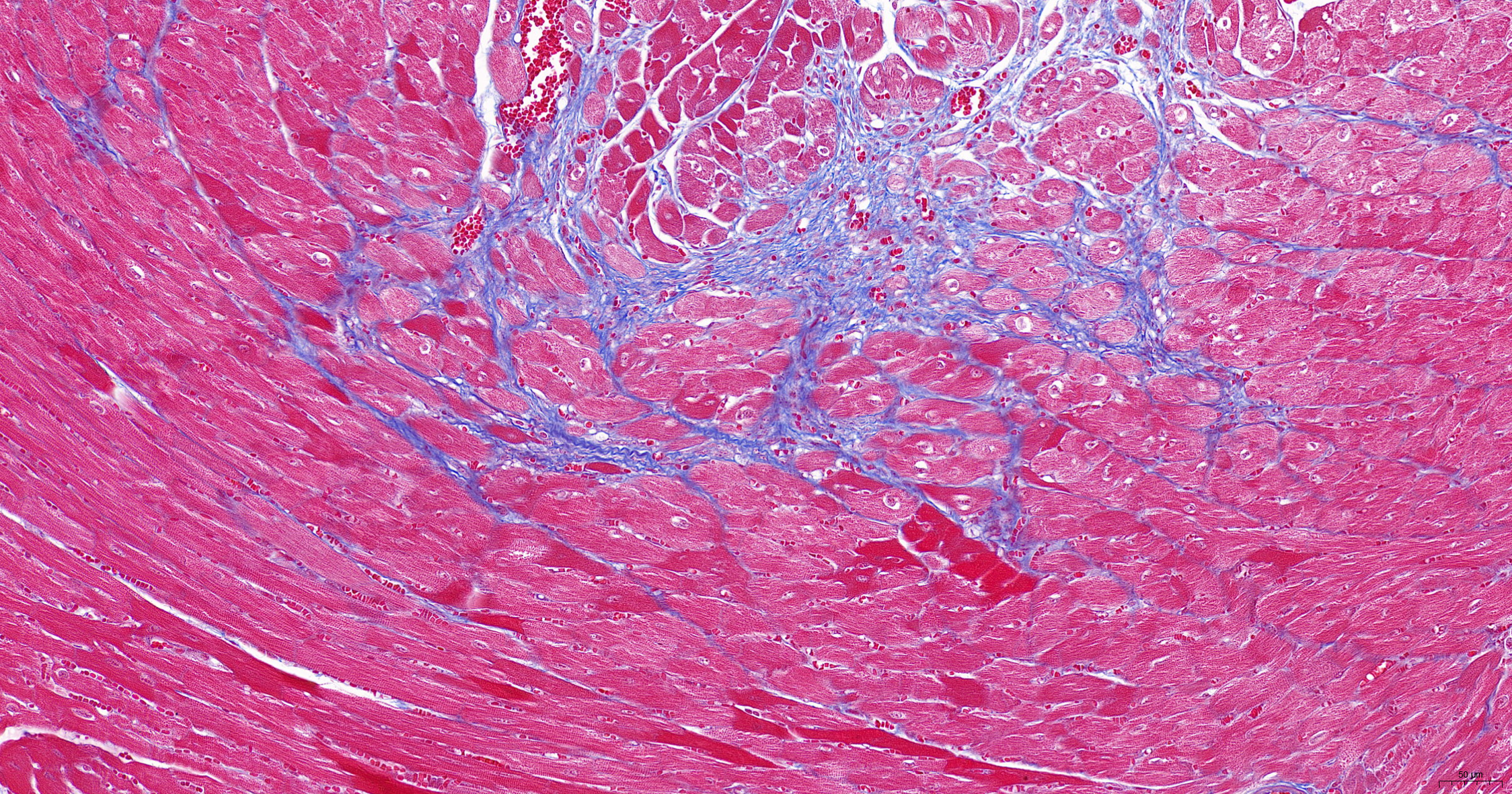

Supplement: S1 File — (ZIP) [file pone.0334880.s001.zip › Supporting information files20251008/Data set for Figure 4/Fig4 A-B/Representative Masson staining images of each group/IP ISO 5mg-kg (2).tif]

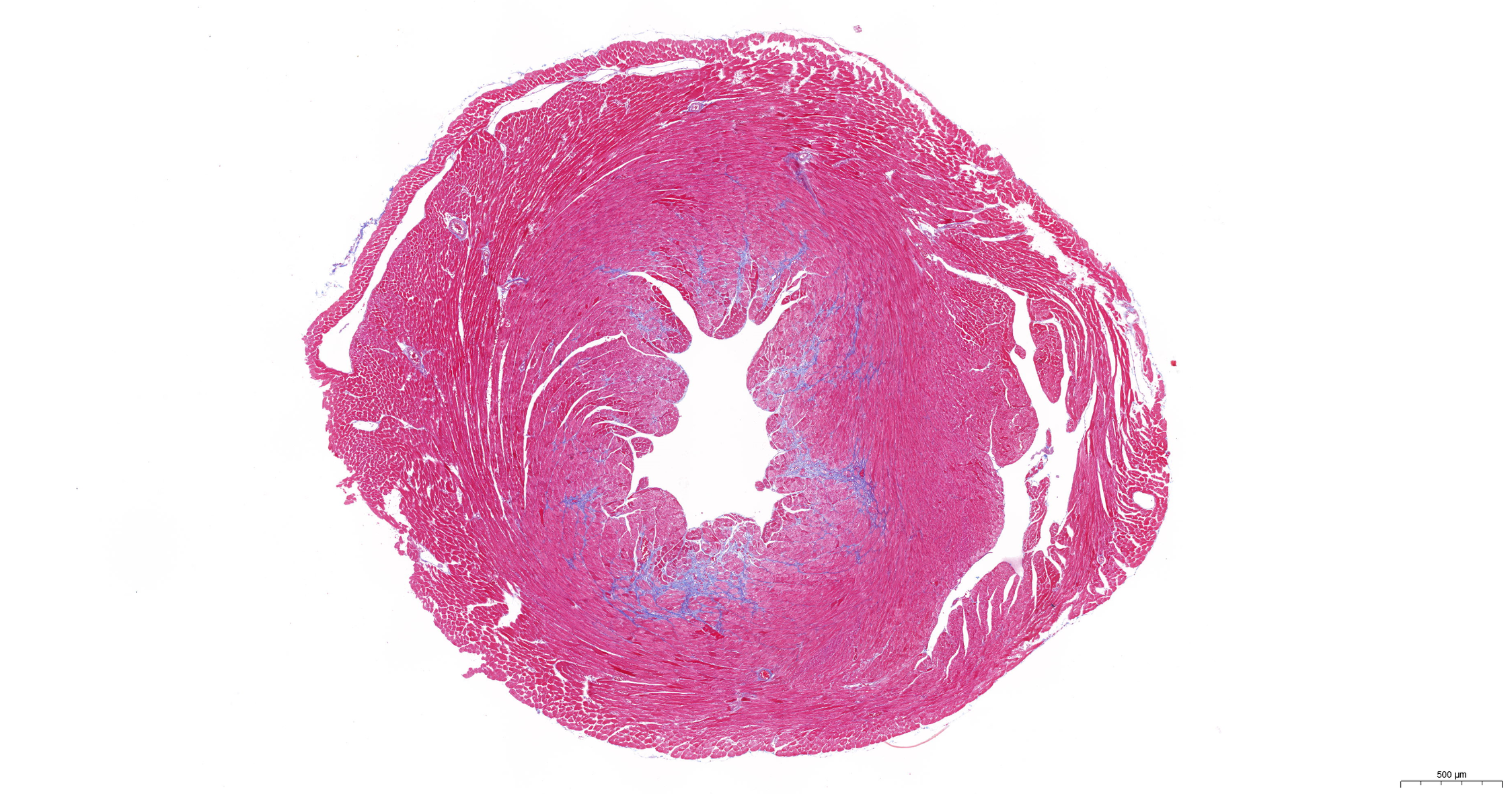

Supplement: S1 File — (ZIP) [file pone.0334880.s001.zip › Supporting information files20251008/Data set for Figure 4/Fig4 A-B/Representative Masson staining images of each group/IP ISO 5mg-kg.tif]

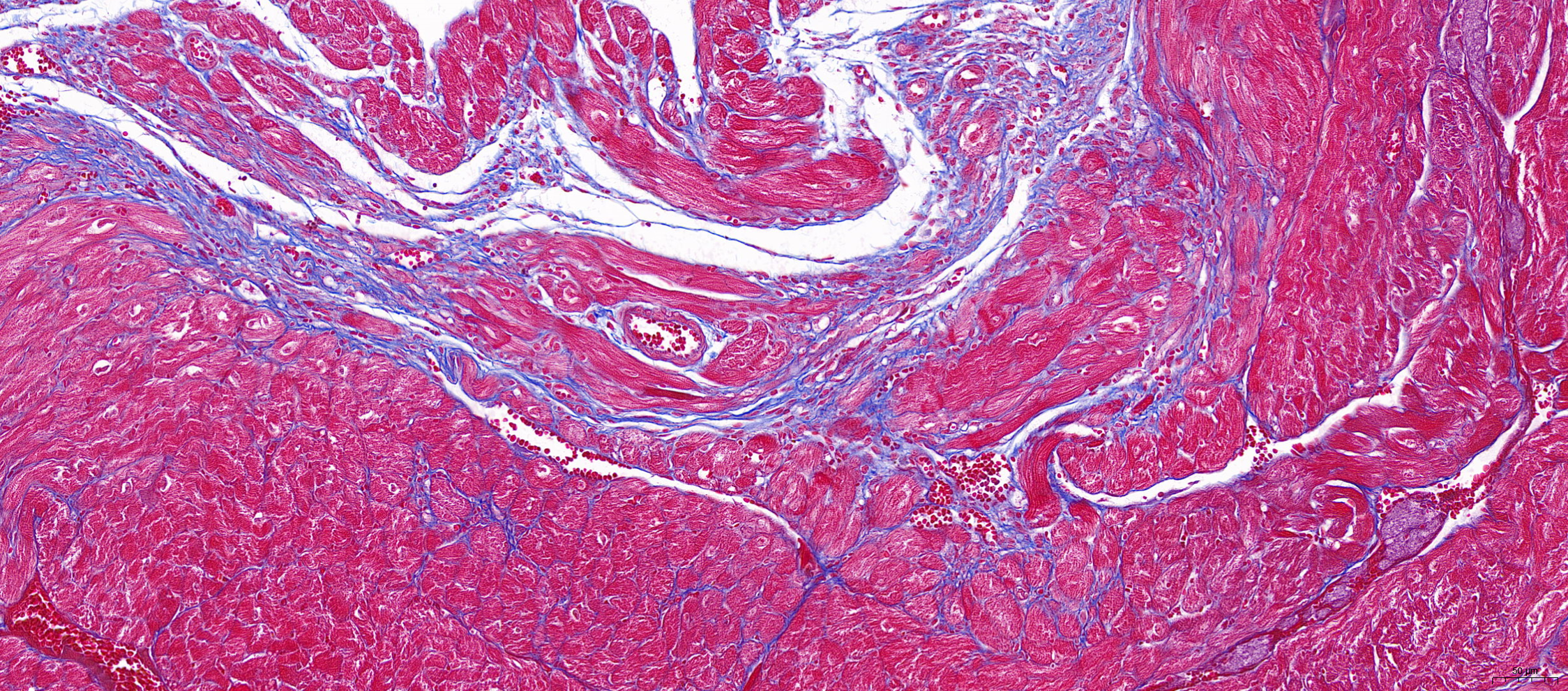

Supplement: S1 File — (ZIP) [file pone.0334880.s001.zip › Supporting information files20251008/Data set for Figure 4/Fig4 A-B/Representative Masson staining images of each group/IP ISO 60mg-kg (2).tif]

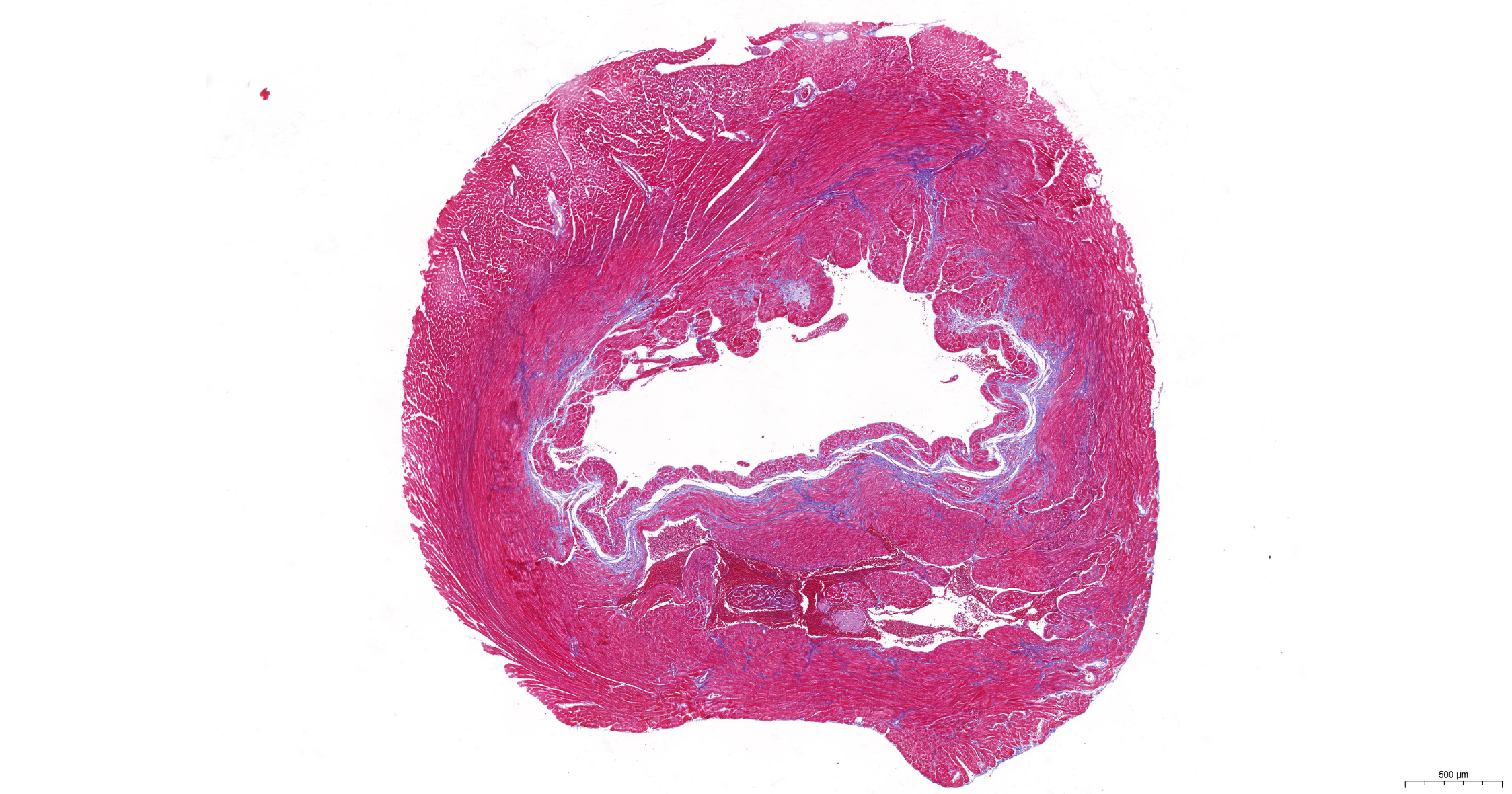

Supplement: S1 File — (ZIP) [file pone.0334880.s001.zip › Supporting information files20251008/Data set for Figure 4/Fig4 A-B/Representative Masson staining images of each group/IP ISO 60mg-kg.tif]

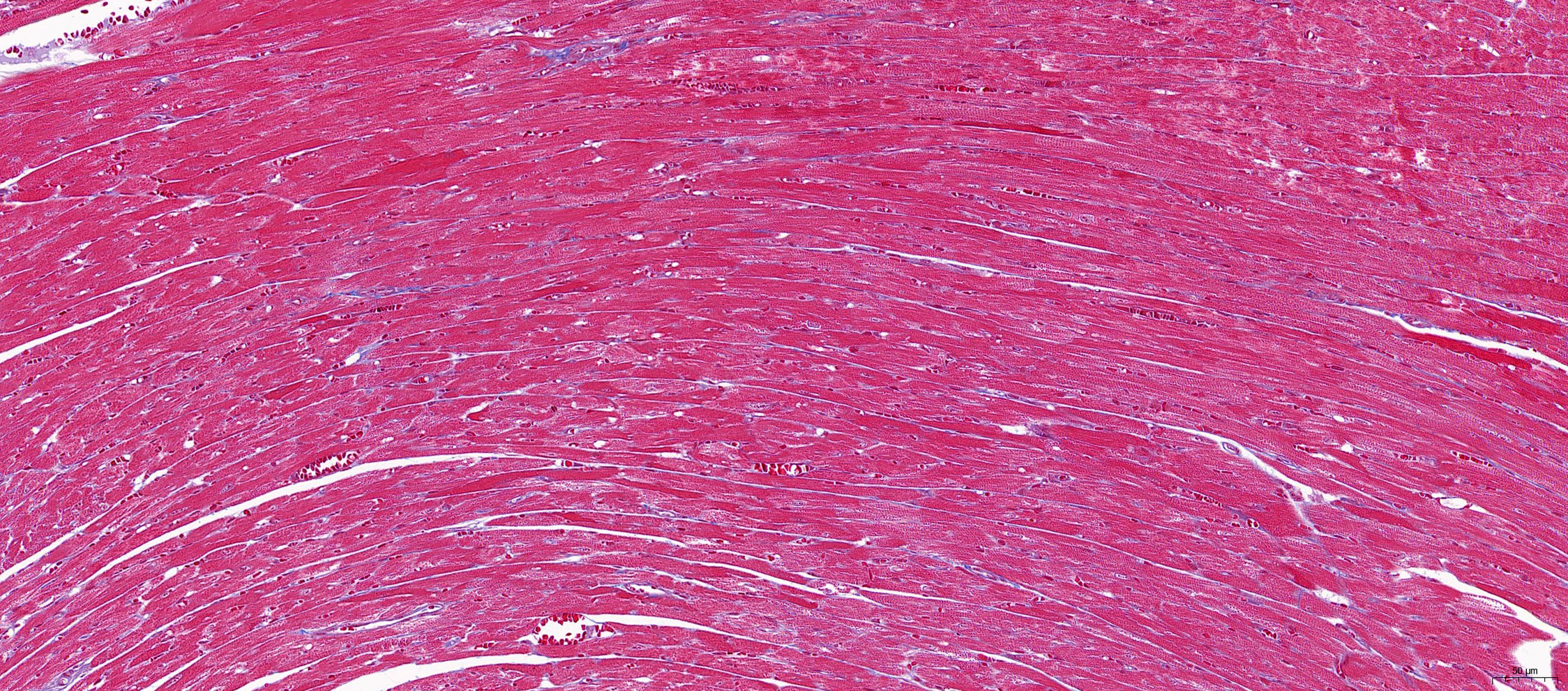

Supplement: S1 File — (ZIP) [file pone.0334880.s001.zip › Supporting information files20251008/Data set for Figure 4/Fig4 A-B/Representative Masson staining images of each group/IP NS (2).tif]

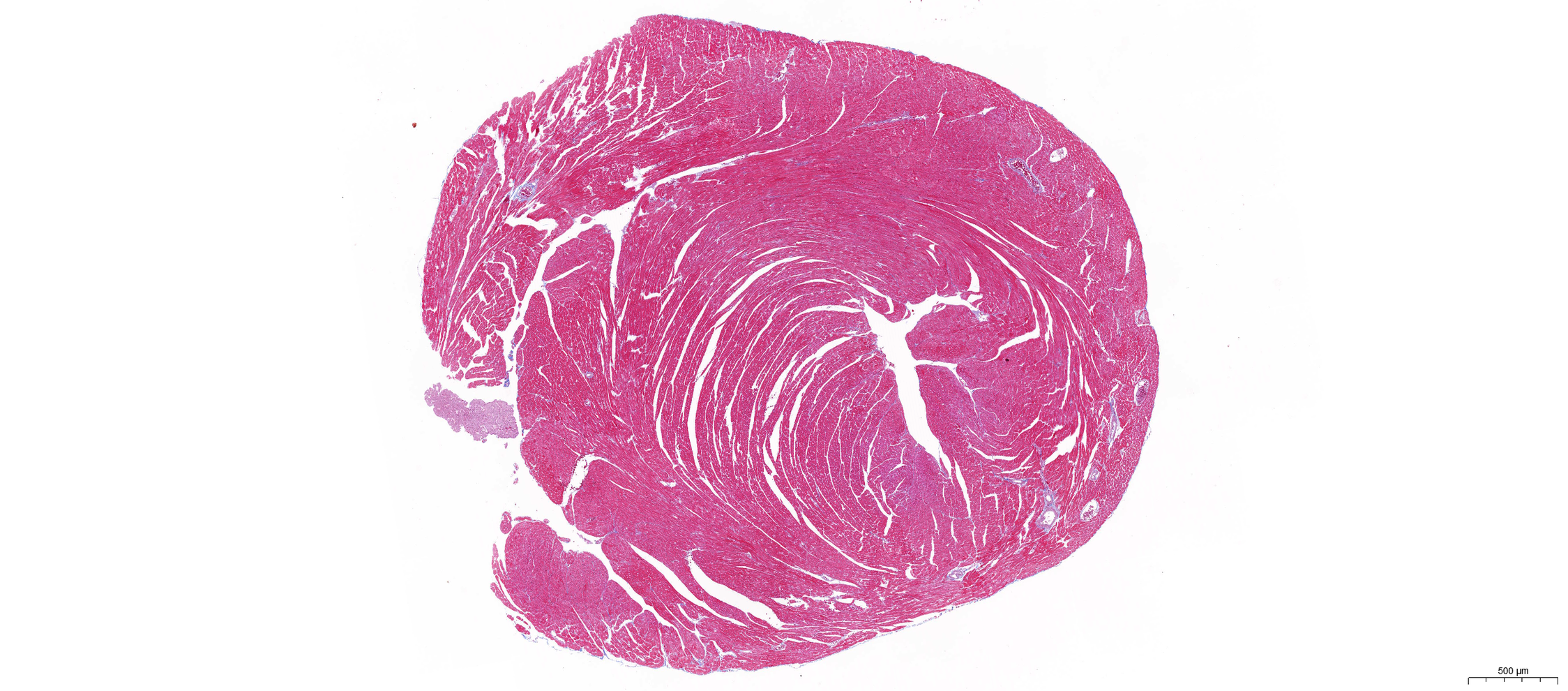

Supplement: S1 File — (ZIP) [file pone.0334880.s001.zip › Supporting information files20251008/Data set for Figure 4/Fig4 A-B/Representative Masson staining images of each group/IP NS.tif]

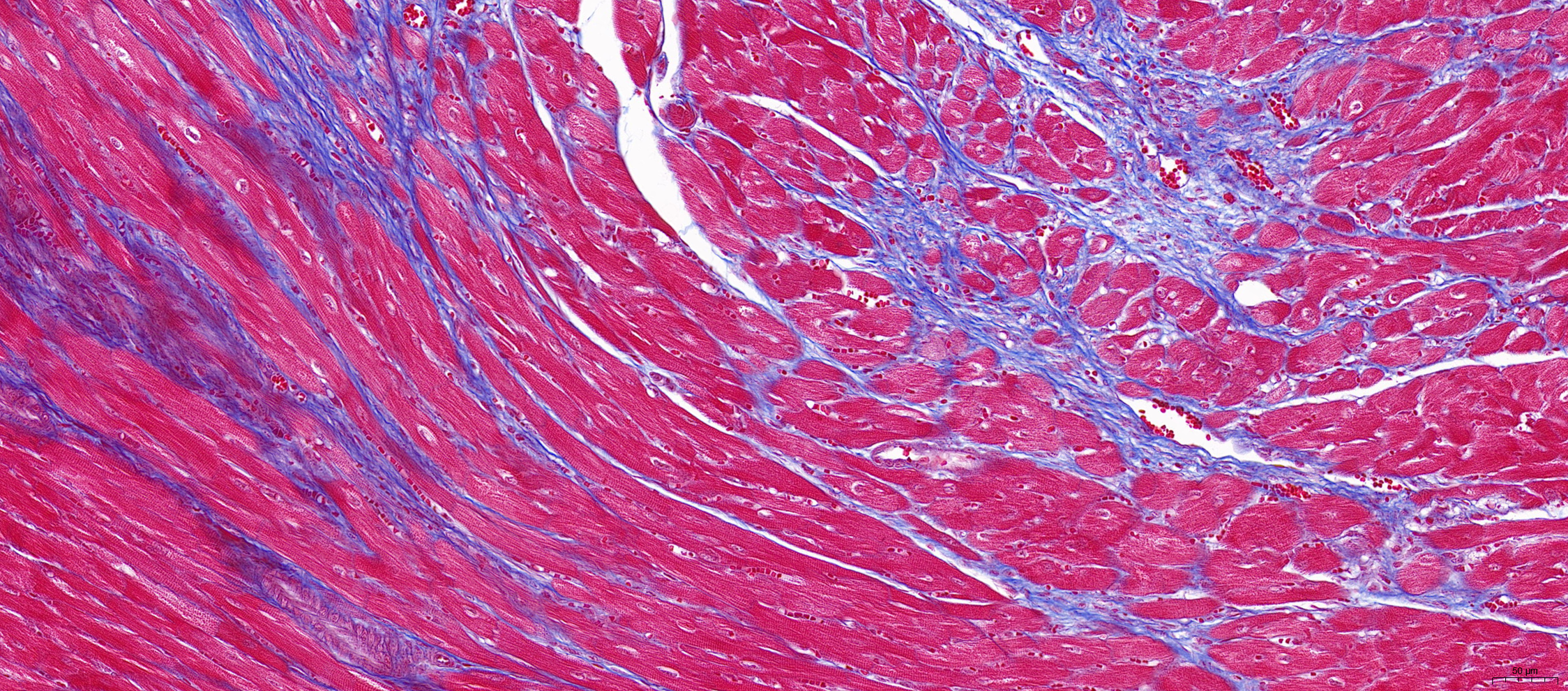

Supplement: S1 File — (ZIP) [file pone.0334880.s001.zip › Supporting information files20251008/Data set for Figure 4/Fig4 A-B/Representative Masson staining images of each group/SC ISO 5mg-kg (2).tif]

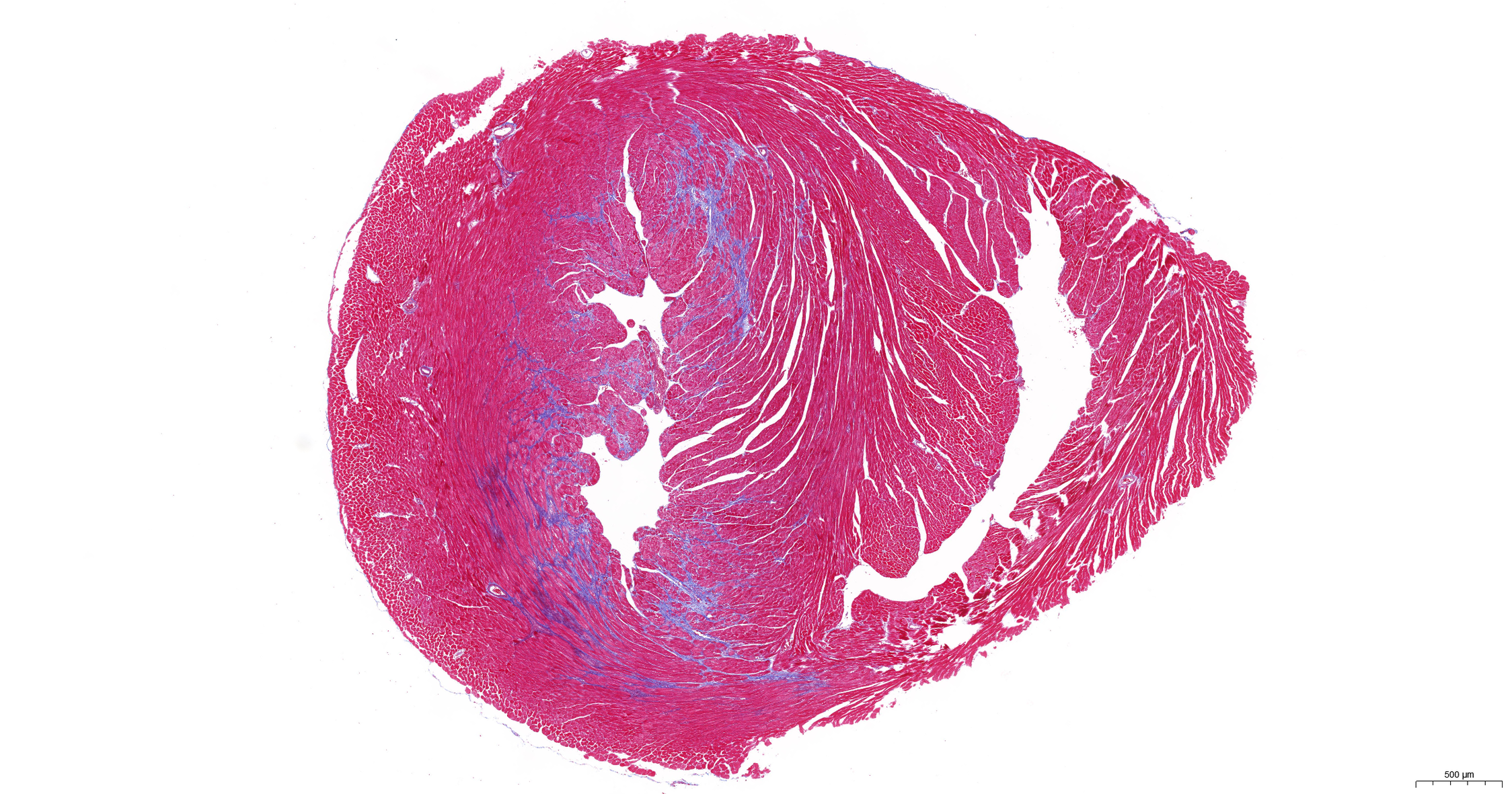

Supplement: S1 File — (ZIP) [file pone.0334880.s001.zip › Supporting information files20251008/Data set for Figure 4/Fig4 A-B/Representative Masson staining images of each group/SC ISO 5mg-kg.tif]

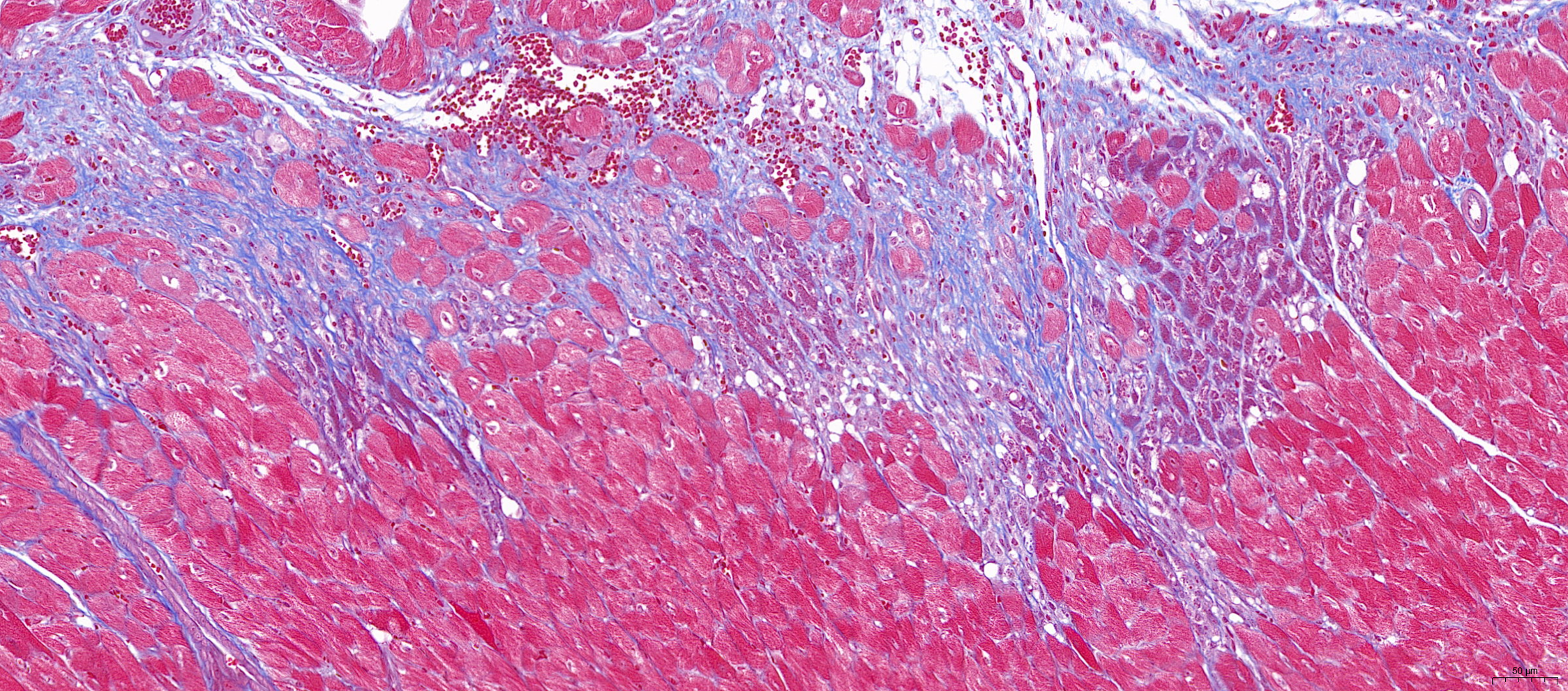

Supplement: S1 File — (ZIP) [file pone.0334880.s001.zip › Supporting information files20251008/Data set for Figure 4/Fig4 A-B/Representative Masson staining images of each group/SC ISO 60mg-kg (2).tif]

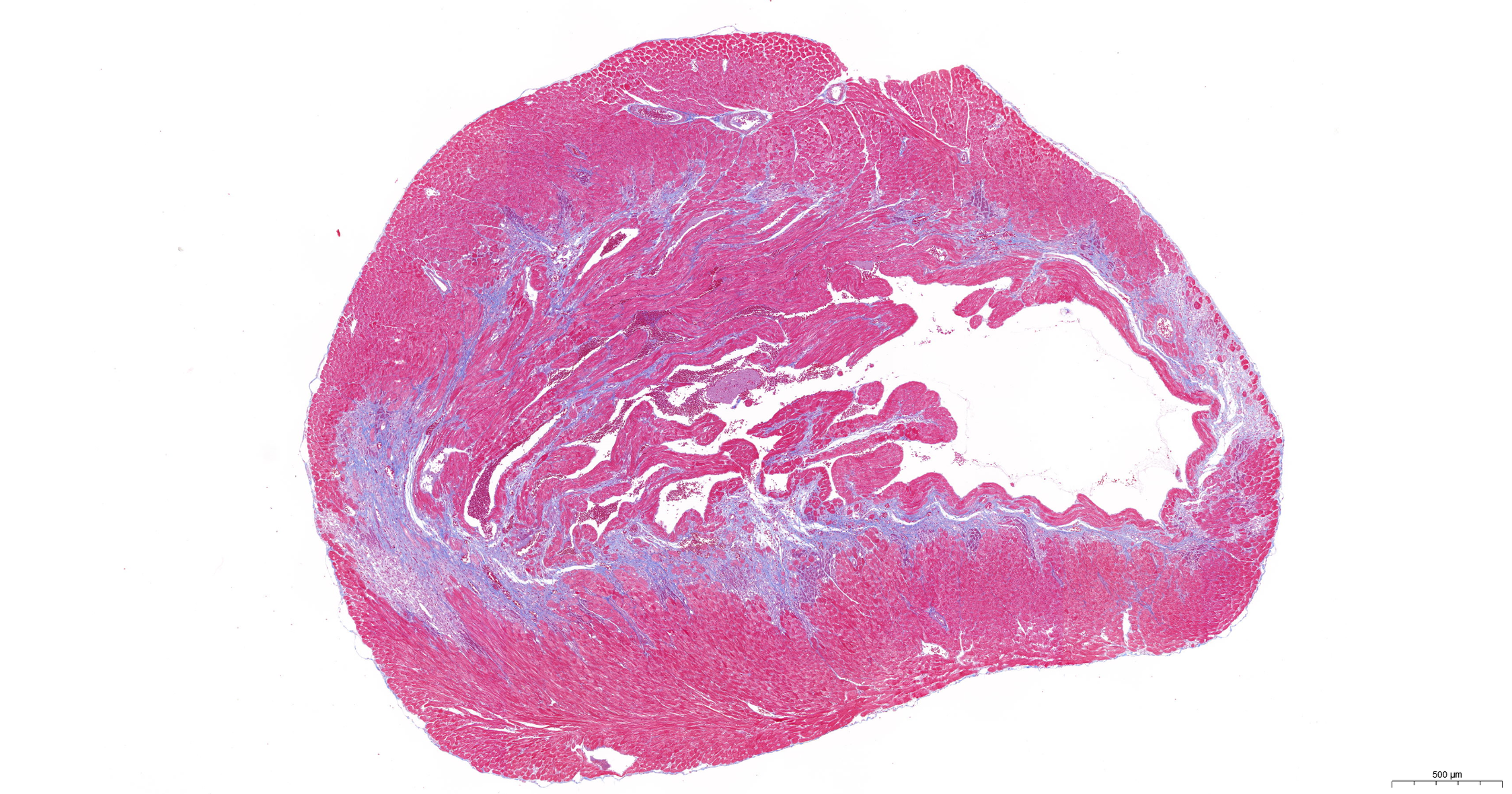

Supplement: S1 File — (ZIP) [file pone.0334880.s001.zip › Supporting information files20251008/Data set for Figure 4/Fig4 A-B/Representative Masson staining images of each group/SC ISO 60mg-kg.tif]

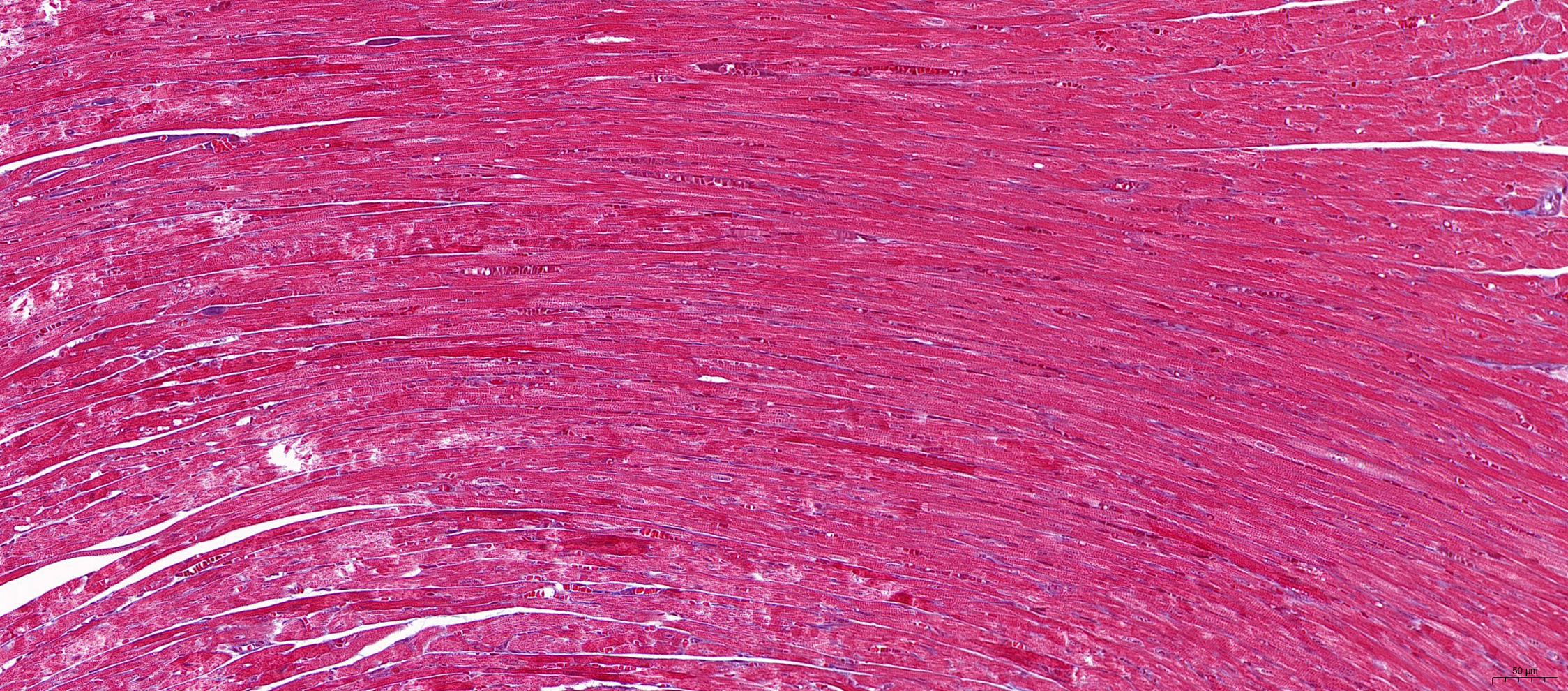

Supplement: S1 File — (ZIP) [file pone.0334880.s001.zip › Supporting information files20251008/Data set for Figure 4/Fig4 A-B/Representative Masson staining images of each group/SC NS (2).tif]

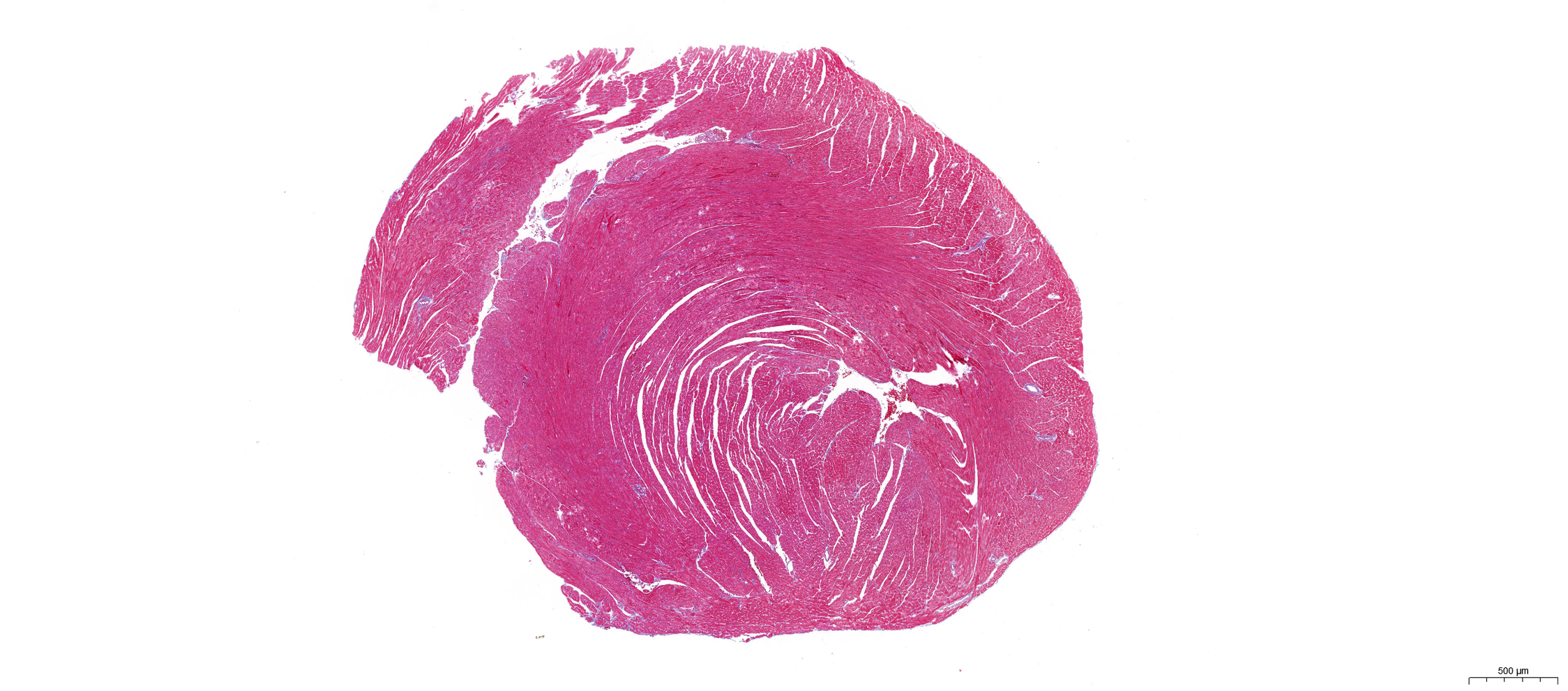

Supplement: S1 File — (ZIP) [file pone.0334880.s001.zip › Supporting information files20251008/Data set for Figure 4/Fig4 A-B/Representative Masson staining images of each group/SC NS.tif]

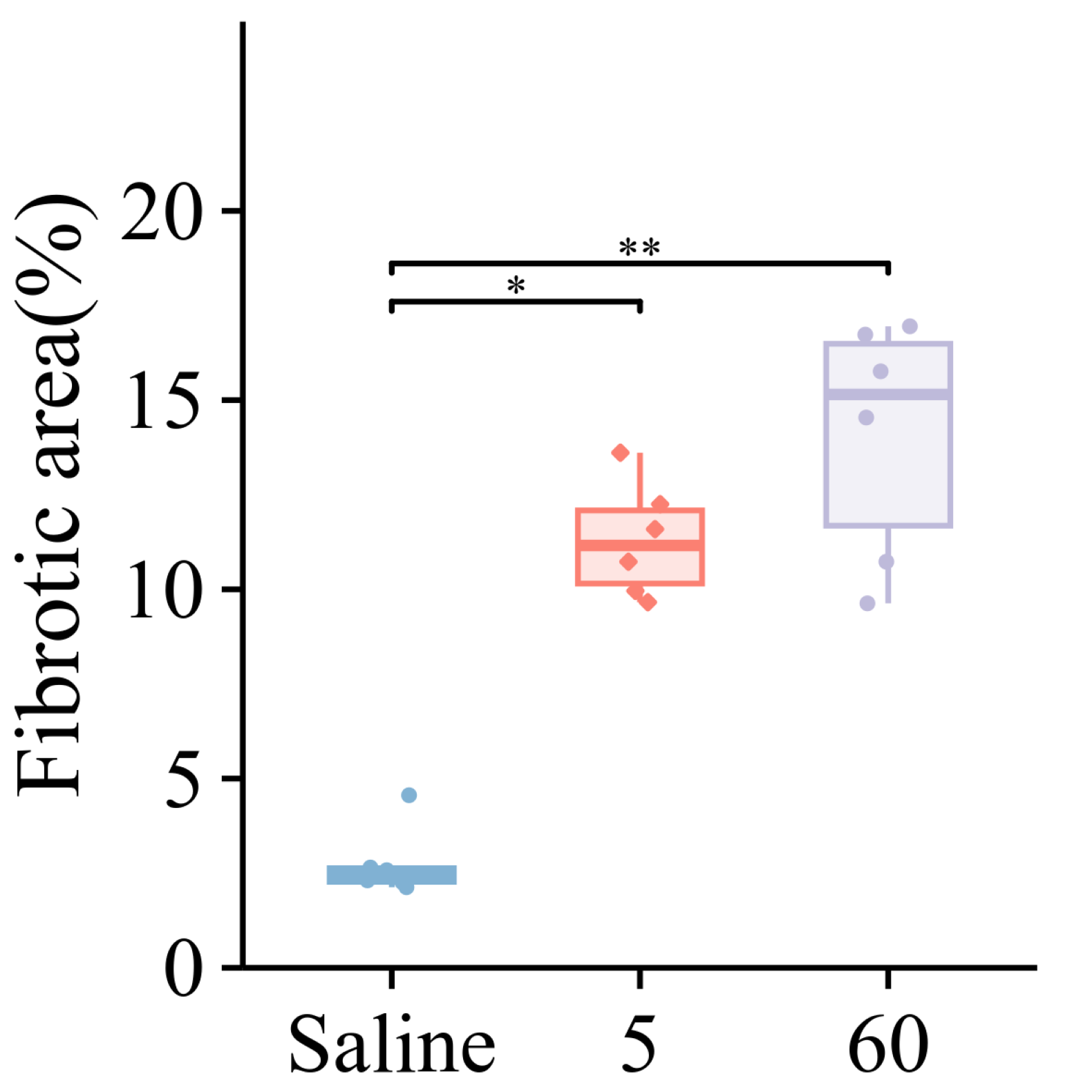

Supplement: S1 File — (ZIP) [file pone.0334880.s001.zip › Supporting information files20251008/Data set for Figure 4/Fig4 A-B/Statistical chart of myocardial fibrosis area in the IP group.tif]

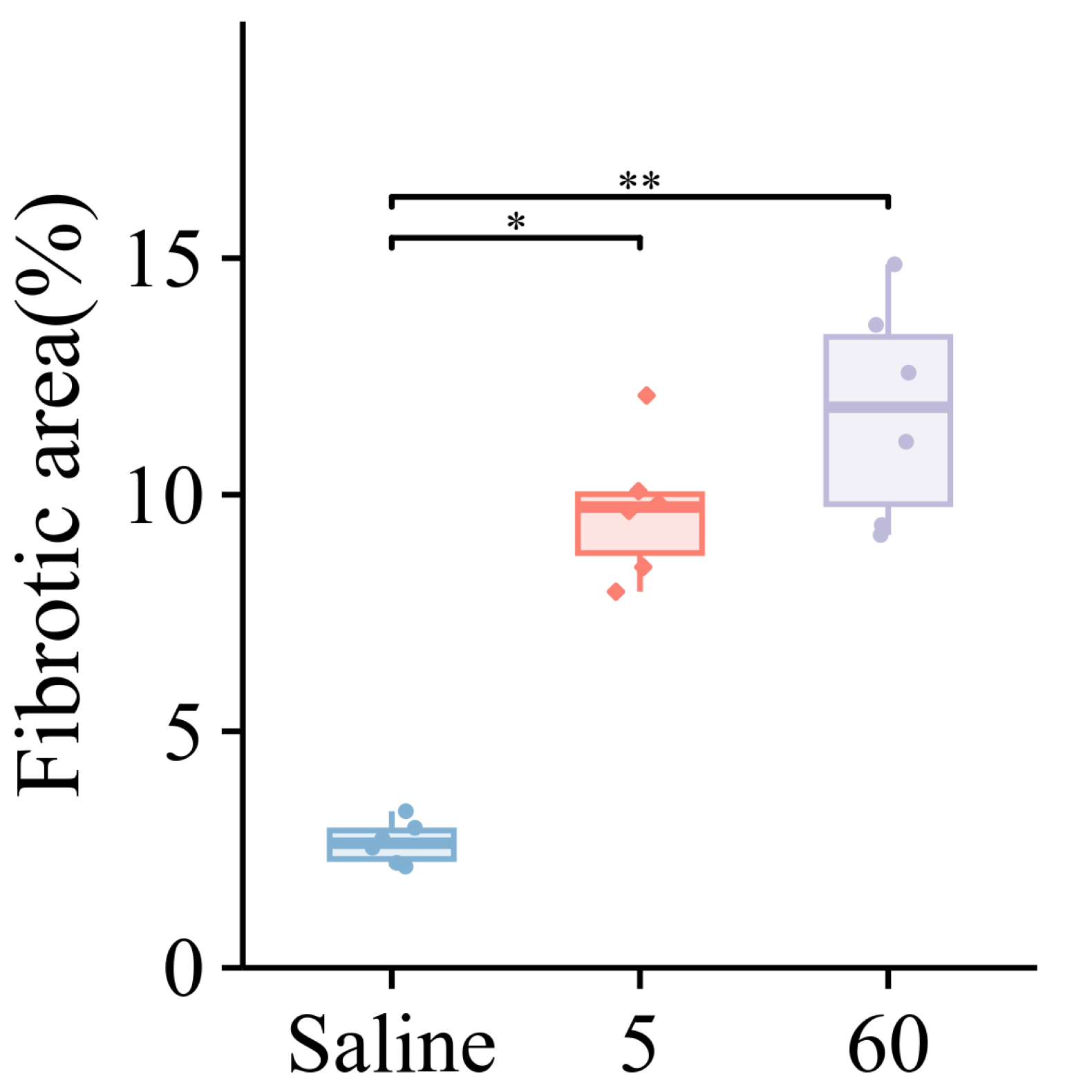

Supplement: S1 File — (ZIP) [file pone.0334880.s001.zip › Supporting information files20251008/Data set for Figure 4/Fig4 A-B/Statistical chart of myocardial fibrosis area in the SC group.tif]

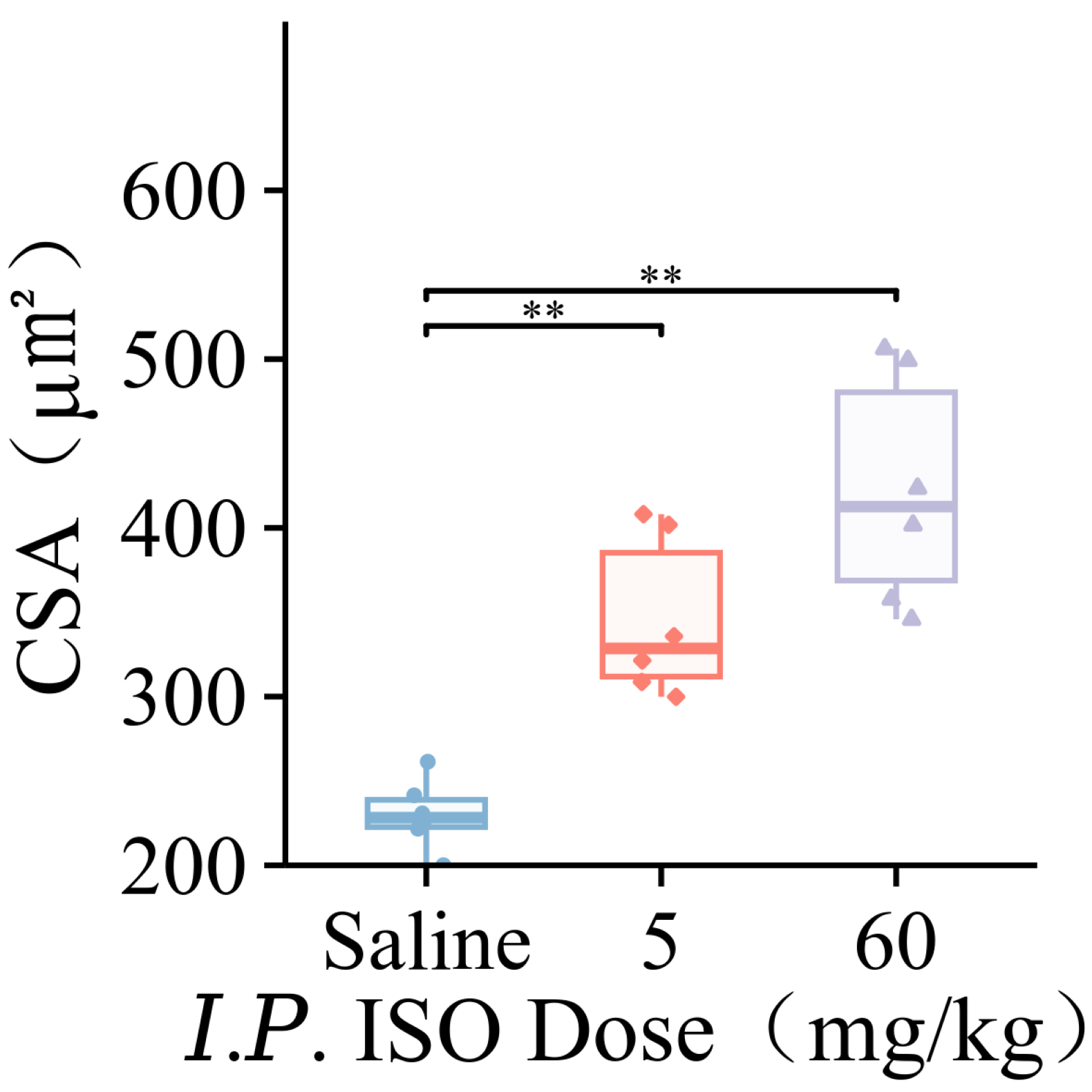

Supplement: S1 File — (ZIP) [file pone.0334880.s001.zip › Supporting information files20251008/Data set for Figure 4/Fig4 C-D/Box-type statistical chart of cross-sectional area of myocardial cells in the IP group.tif]

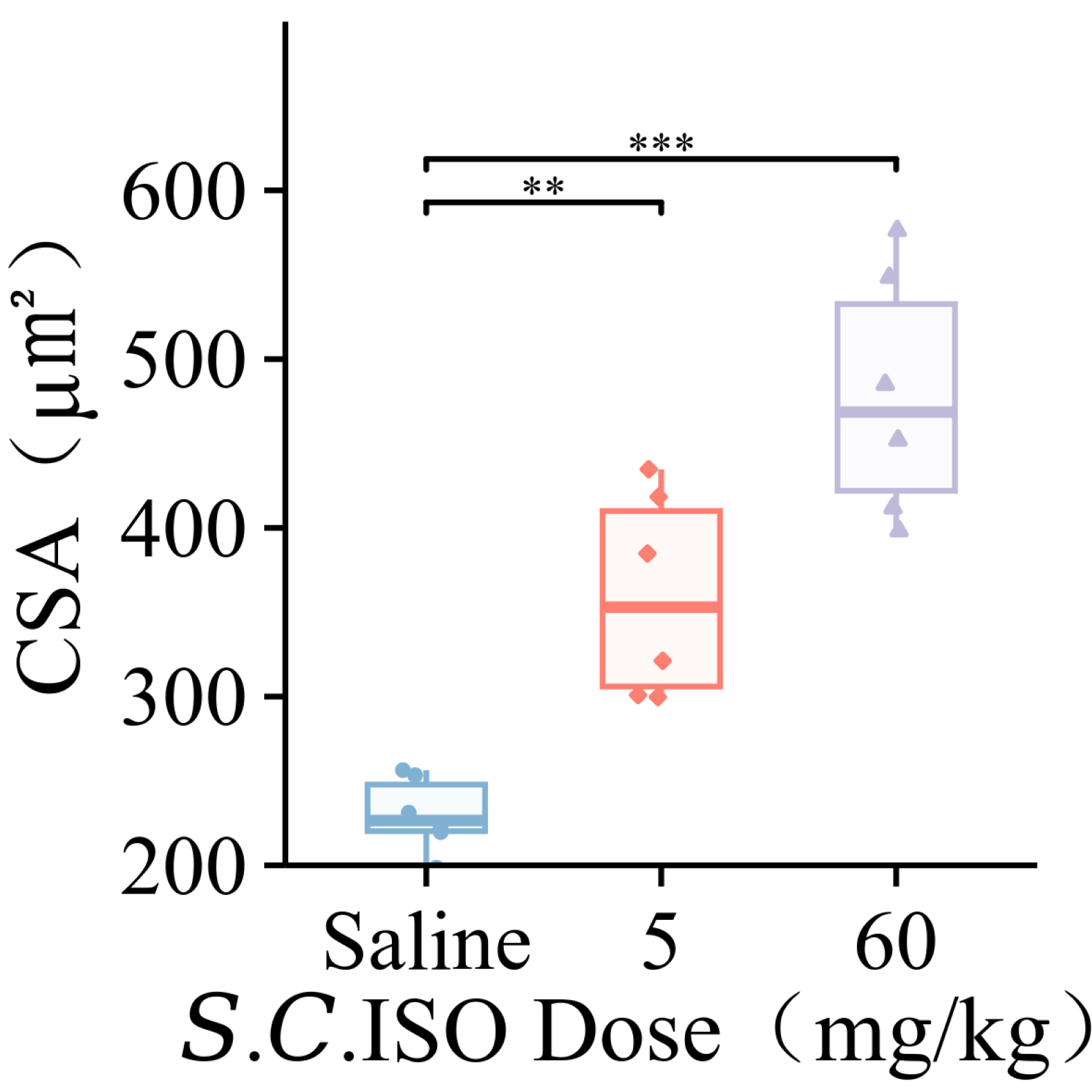

Supplement: S1 File — (ZIP) [file pone.0334880.s001.zip › Supporting information files20251008/Data set for Figure 4/Fig4 C-D/Box-type statistical chart of cross-sectional area of myocardial cells in the SC group.tif]

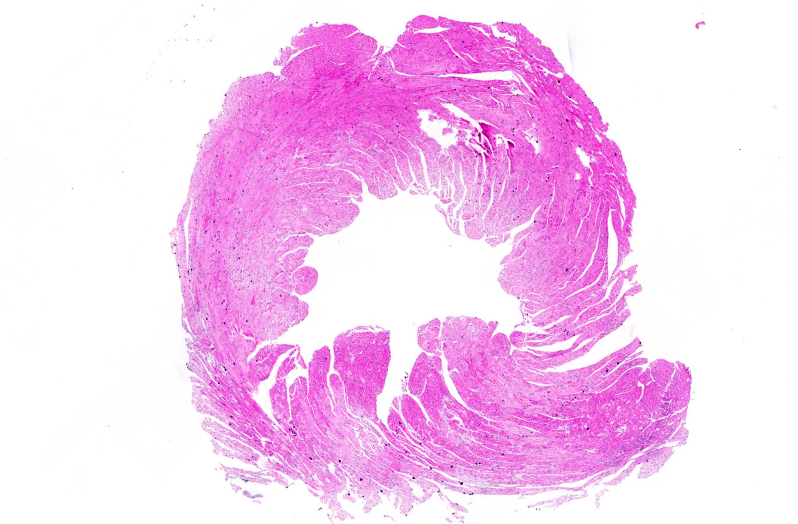

Supplement: S1 File — (ZIP) [file pone.0334880.s001.zip › Supporting information files20251008/Data set for Figure 4/Fig4 C-D/Representative HE staining images of each group/IP ISO 5mg-kg-2.tif]

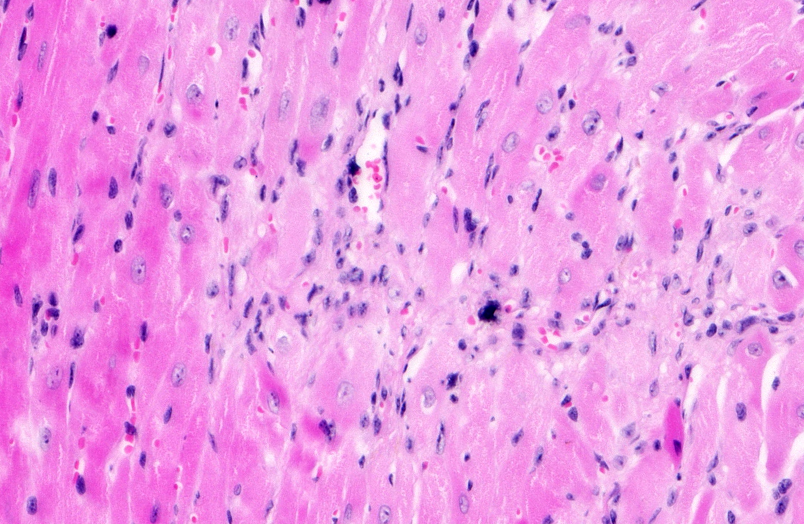

Supplement: S1 File — (ZIP) [file pone.0334880.s001.zip › Supporting information files20251008/Data set for Figure 4/Fig4 C-D/Representative HE staining images of each group/IP ISO 5mg-kg.tif]

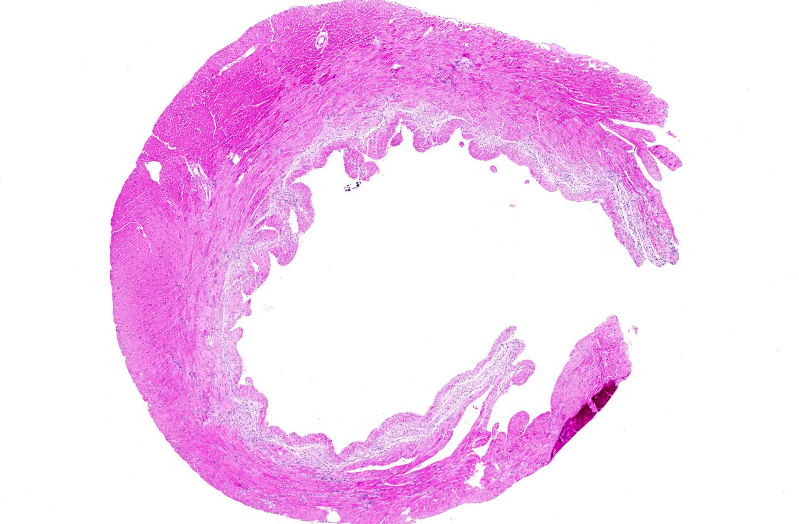

Supplement: S1 File — (ZIP) [file pone.0334880.s001.zip › Supporting information files20251008/Data set for Figure 4/Fig4 C-D/Representative HE staining images of each group/IP ISO 60mg-kg-2.tif]

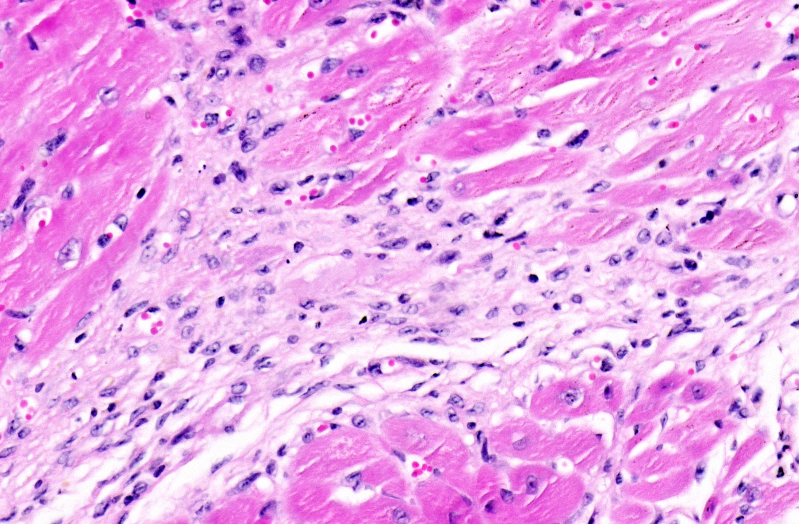

Supplement: S1 File — (ZIP) [file pone.0334880.s001.zip › Supporting information files20251008/Data set for Figure 4/Fig4 C-D/Representative HE staining images of each group/IP ISO 60mg-kg.tif]

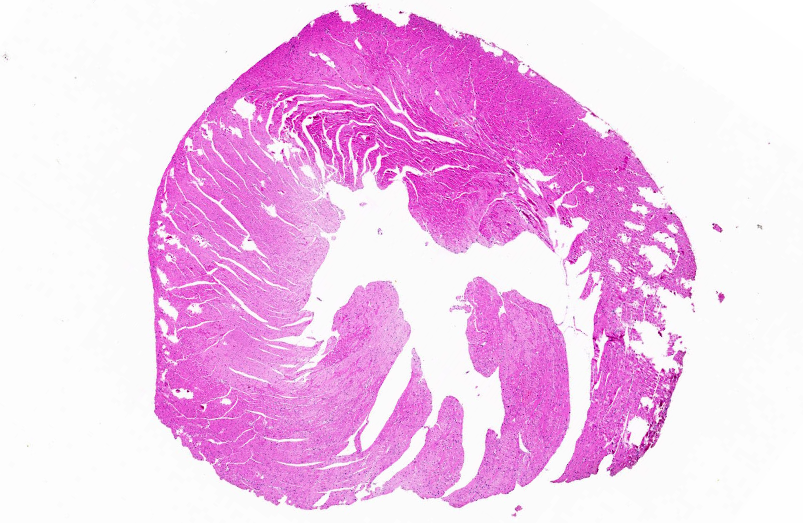

Supplement: S1 File — (ZIP) [file pone.0334880.s001.zip › Supporting information files20251008/Data set for Figure 4/Fig4 C-D/Representative HE staining images of each group/IP NS-2.tif]

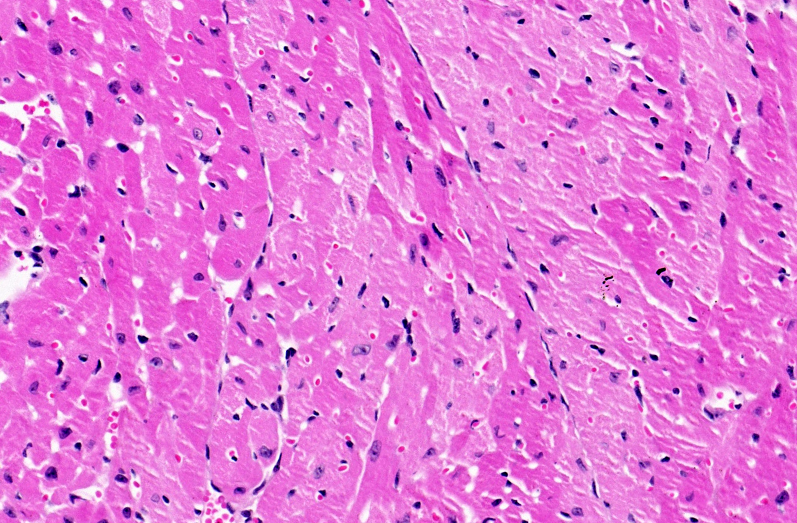

Supplement: S1 File — (ZIP) [file pone.0334880.s001.zip › Supporting information files20251008/Data set for Figure 4/Fig4 C-D/Representative HE staining images of each group/IP NS.tif]

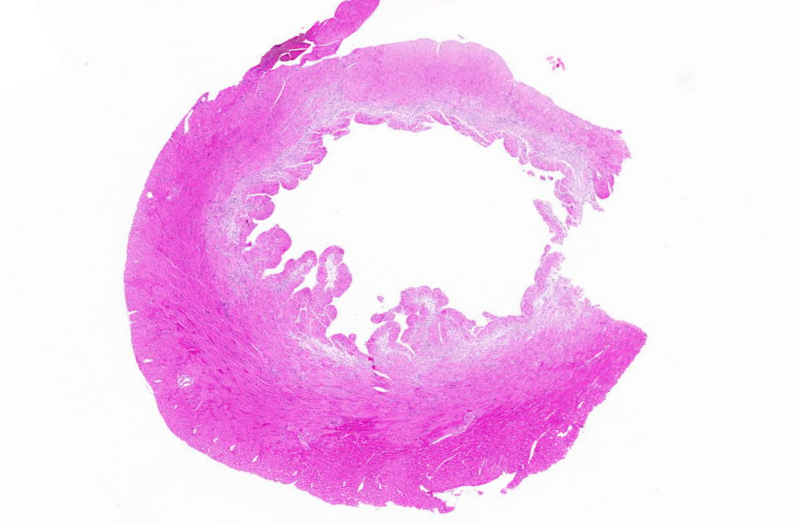

Supplement: S1 File — (ZIP) [file pone.0334880.s001.zip › Supporting information files20251008/Data set for Figure 4/Fig4 C-D/Representative HE staining images of each group/SC ISO 5mg-kg-2.tif]

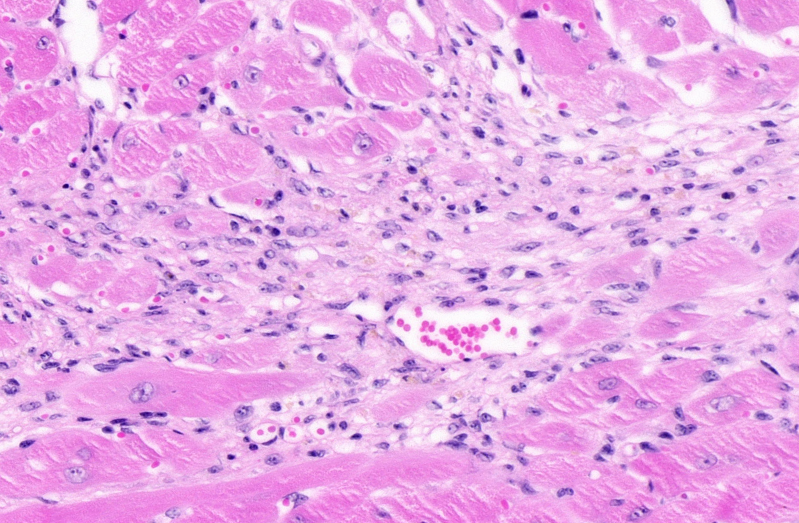

Supplement: S1 File — (ZIP) [file pone.0334880.s001.zip › Supporting information files20251008/Data set for Figure 4/Fig4 C-D/Representative HE staining images of each group/SC ISO 5mg-kg.tif]

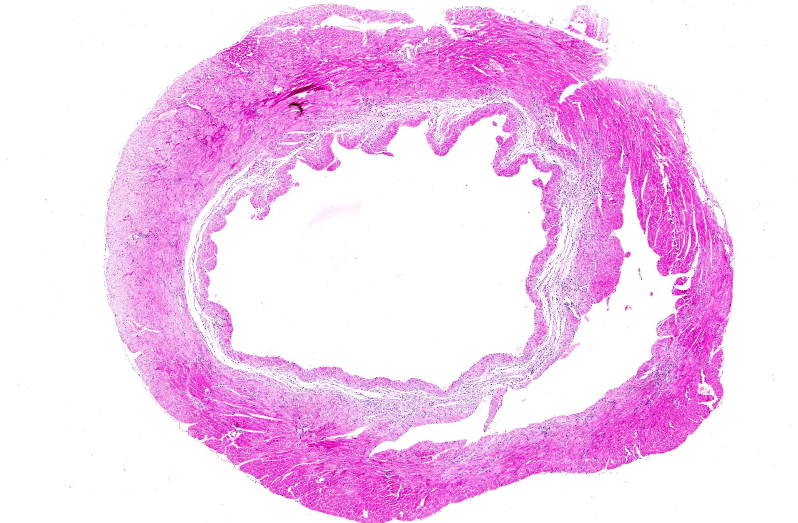

Supplement: S1 File — (ZIP) [file pone.0334880.s001.zip › Supporting information files20251008/Data set for Figure 4/Fig4 C-D/Representative HE staining images of each group/SC ISO 60mg-kg-2.tif]

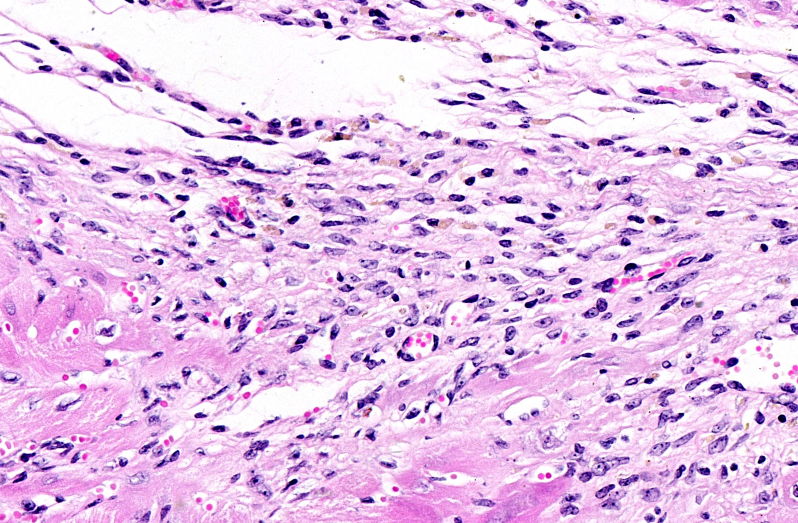

Supplement: S1 File — (ZIP) [file pone.0334880.s001.zip › Supporting information files20251008/Data set for Figure 4/Fig4 C-D/Representative HE staining images of each group/SC ISO 60mg-kg.tif]

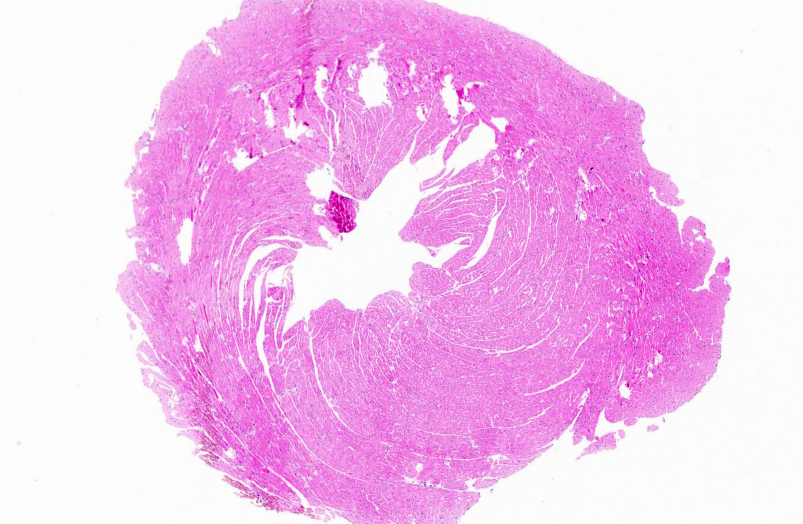

Supplement: S1 File — (ZIP) [file pone.0334880.s001.zip › Supporting information files20251008/Data set for Figure 4/Fig4 C-D/Representative HE staining images of each group/SC NS-2.tif]

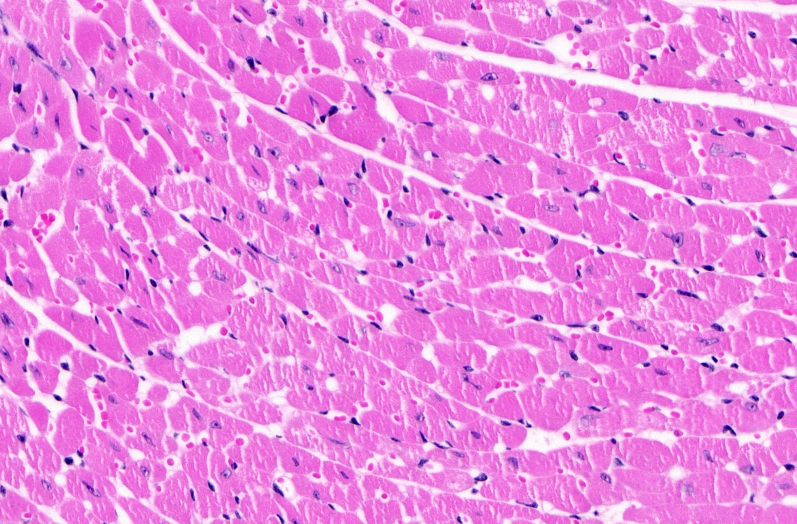

Supplement: S1 File — (ZIP) [file pone.0334880.s001.zip › Supporting information files20251008/Data set for Figure 4/Fig4 C-D/Representative HE staining images of each group/SC NS.tif]

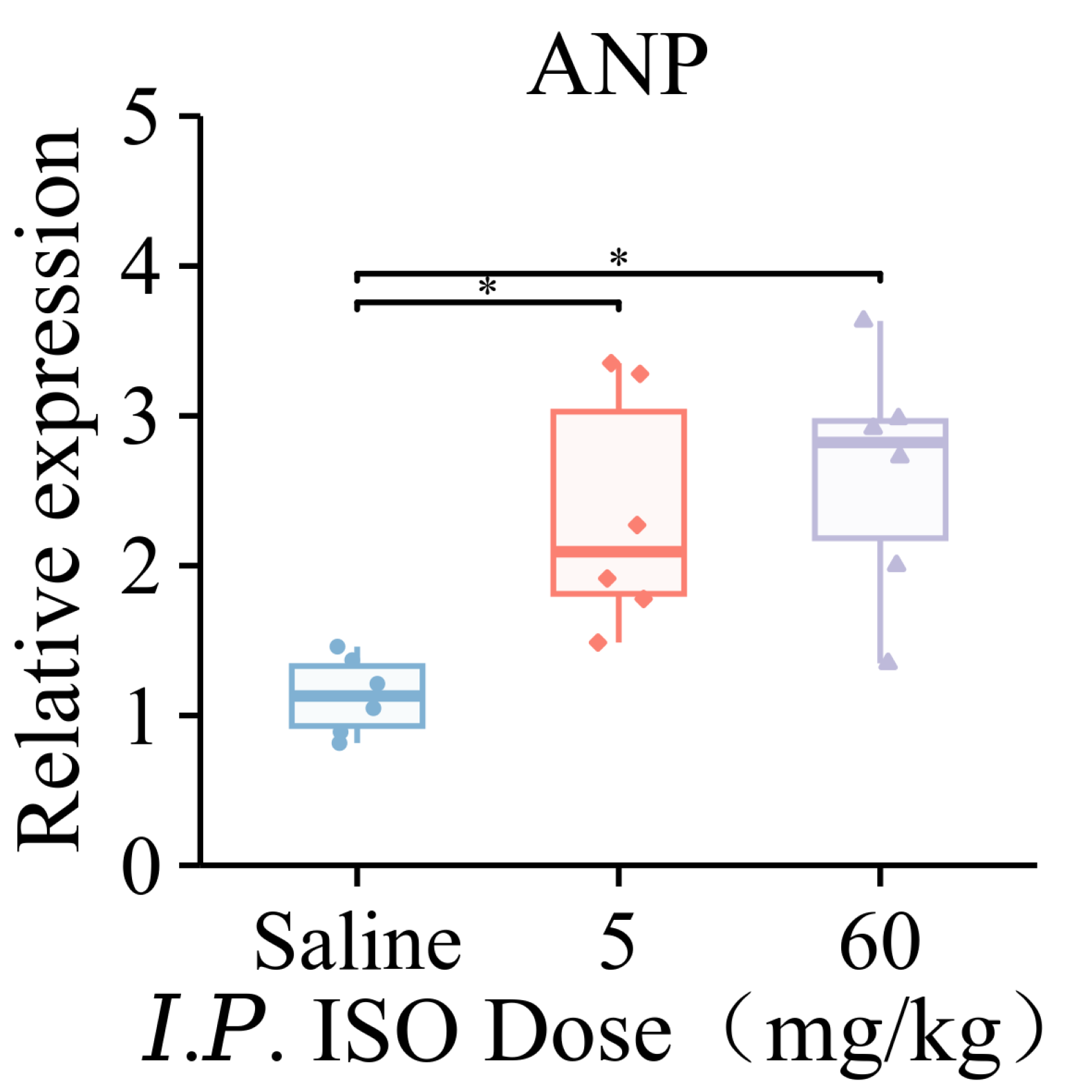

Supplement: S1 File — (ZIP) [file pone.0334880.s001.zip › Supporting information files20251008/Data set for Figure 5/Fig5A-ANP/Box chart of ANP expression levels in the IP group.tif]

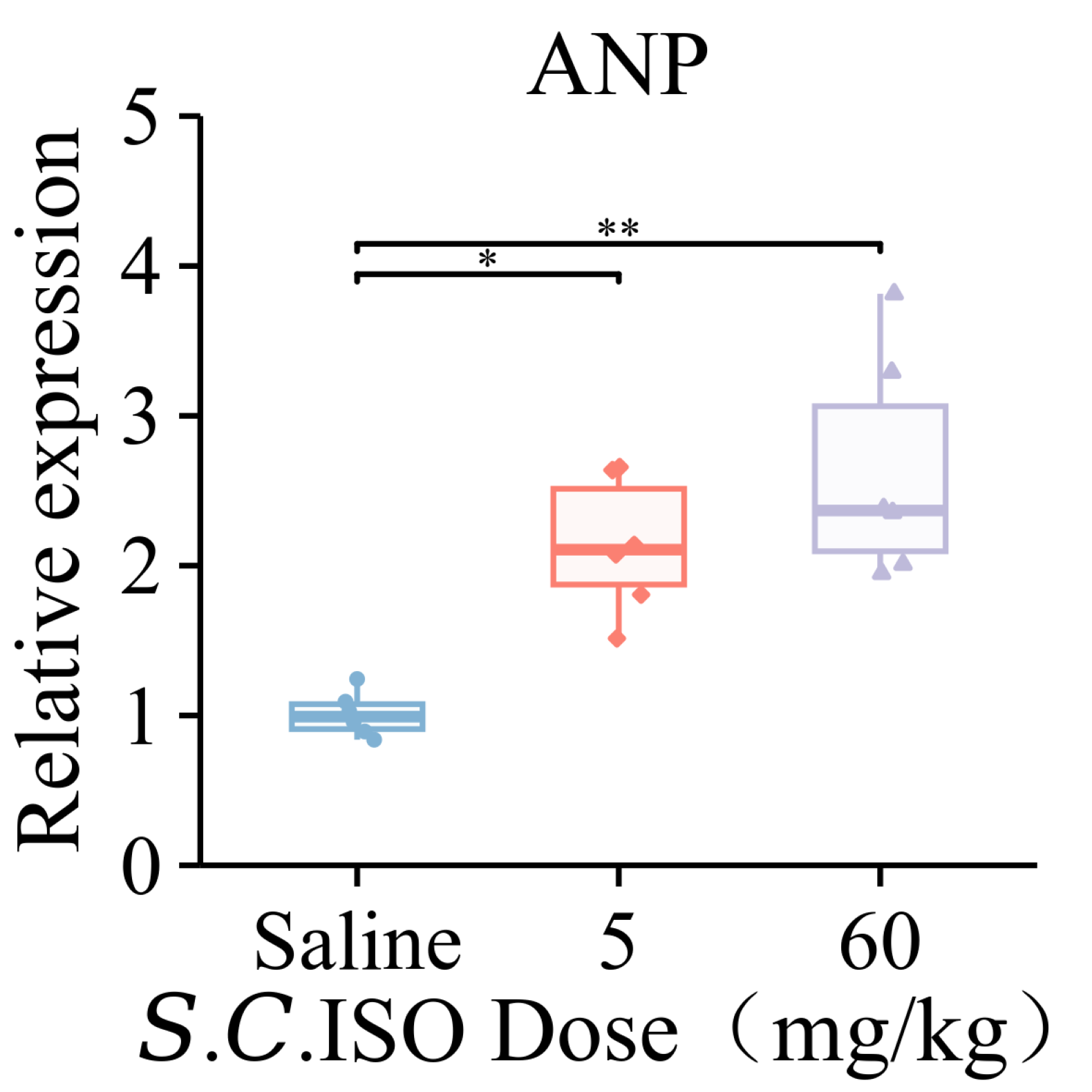

Supplement: S1 File — (ZIP) [file pone.0334880.s001.zip › Supporting information files20251008/Data set for Figure 5/Fig5A-ANP/Box chart of ANP expression levels in the SC group.tif]

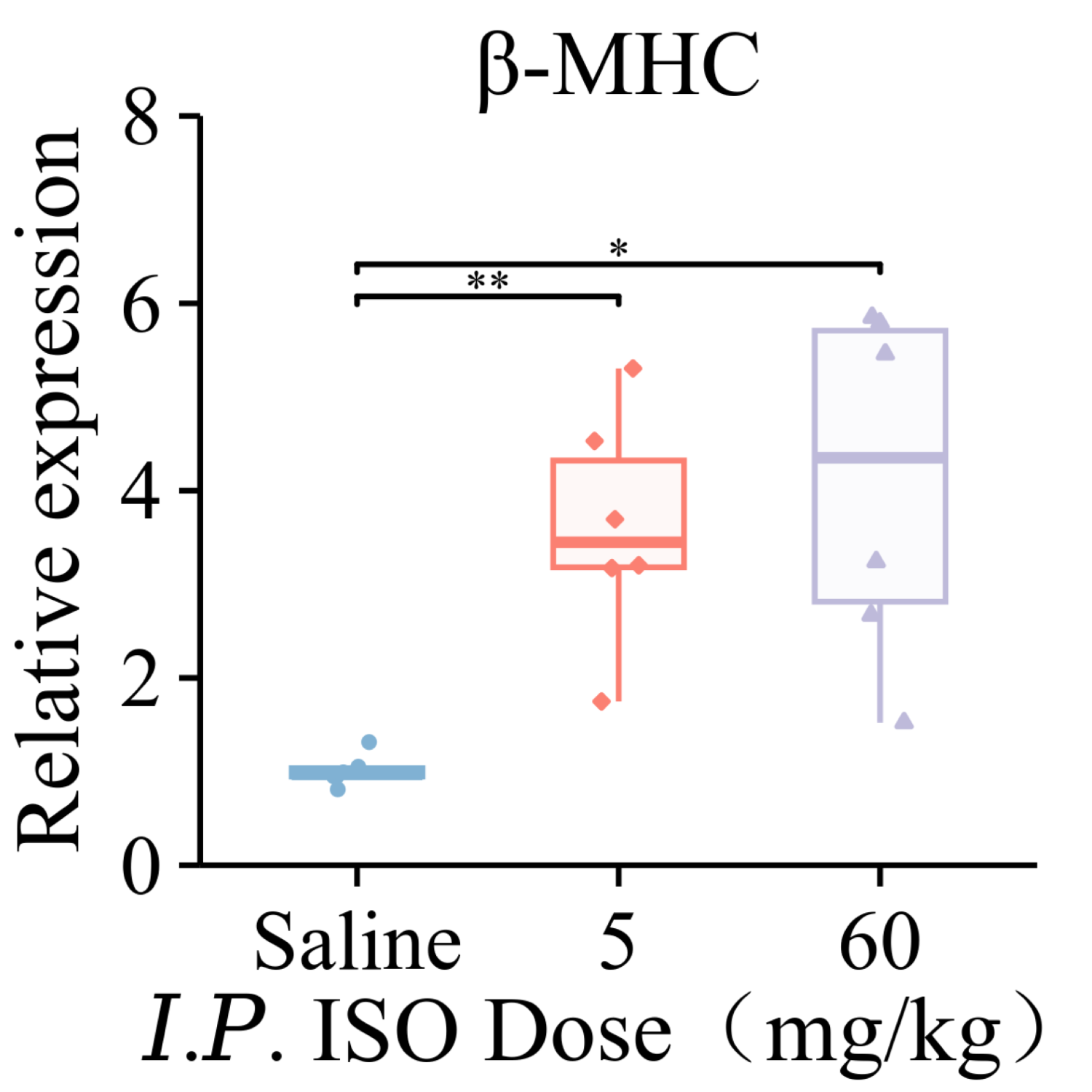

Supplement: S1 File — (ZIP) [file pone.0334880.s001.zip › Supporting information files20251008/Data set for Figure 5/Fig5B-β-MHC/Box chart of β-MHC expression levels in the IP group.tif]

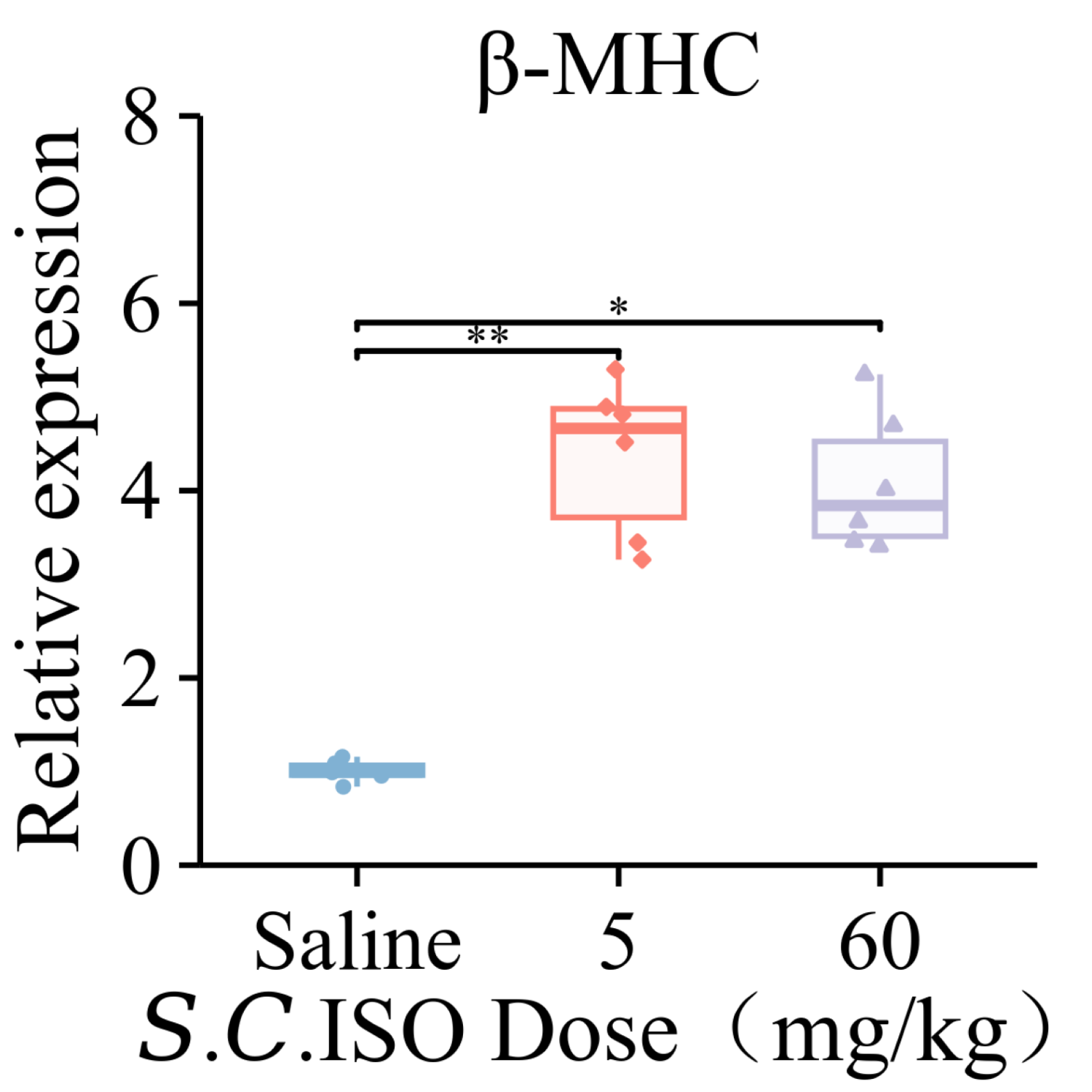

Supplement: S1 File — (ZIP) [file pone.0334880.s001.zip › Supporting information files20251008/Data set for Figure 5/Fig5B-β-MHC/Box chart of β-MHC expression levels in the SC group.tif]

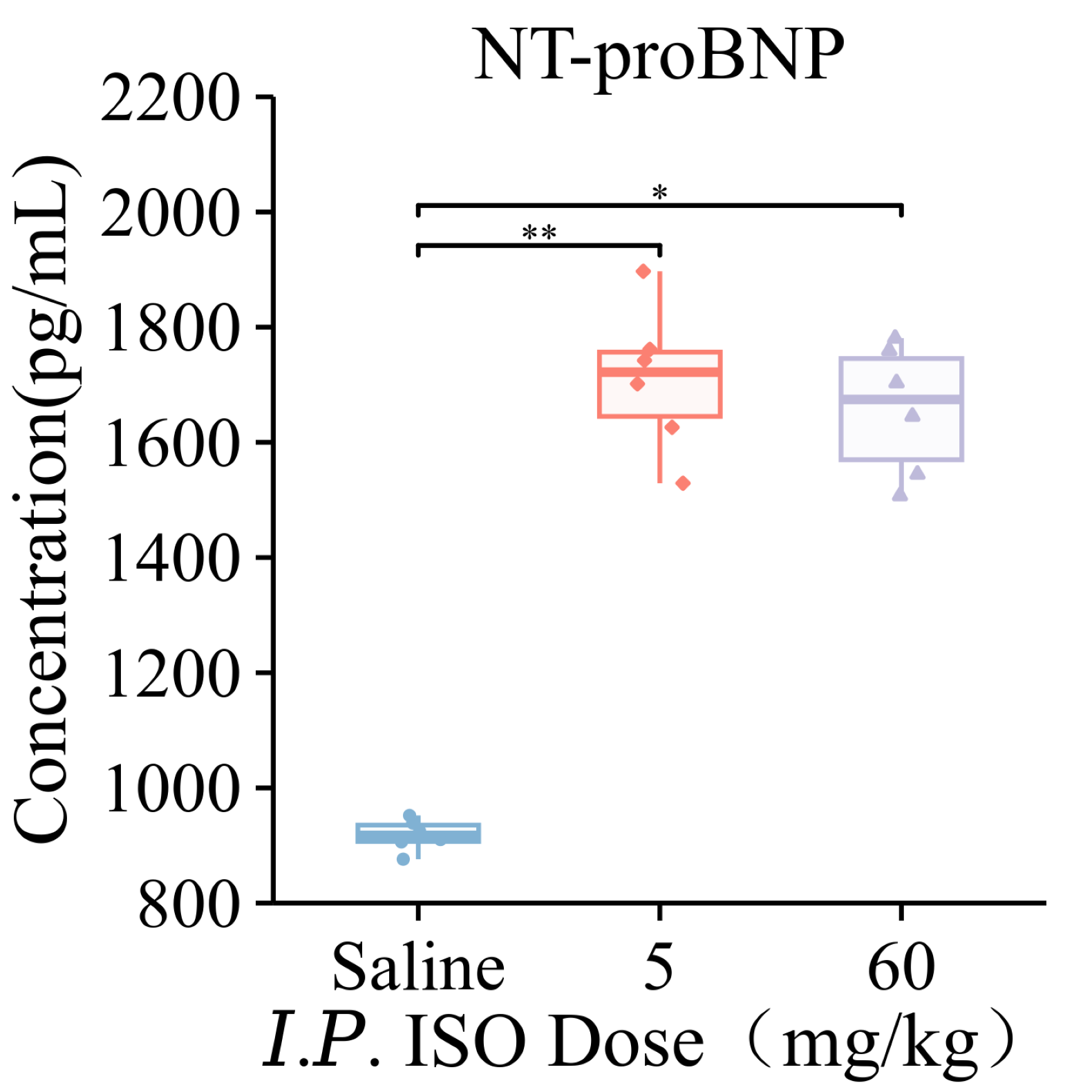

Supplement: S1 File — (ZIP) [file pone.0334880.s001.zip › Supporting information files20251008/Data set for Figure 5/Fig5C-NTpro-BNP/Box chart of NTpro-BNP expression levels in the IP group.tif]

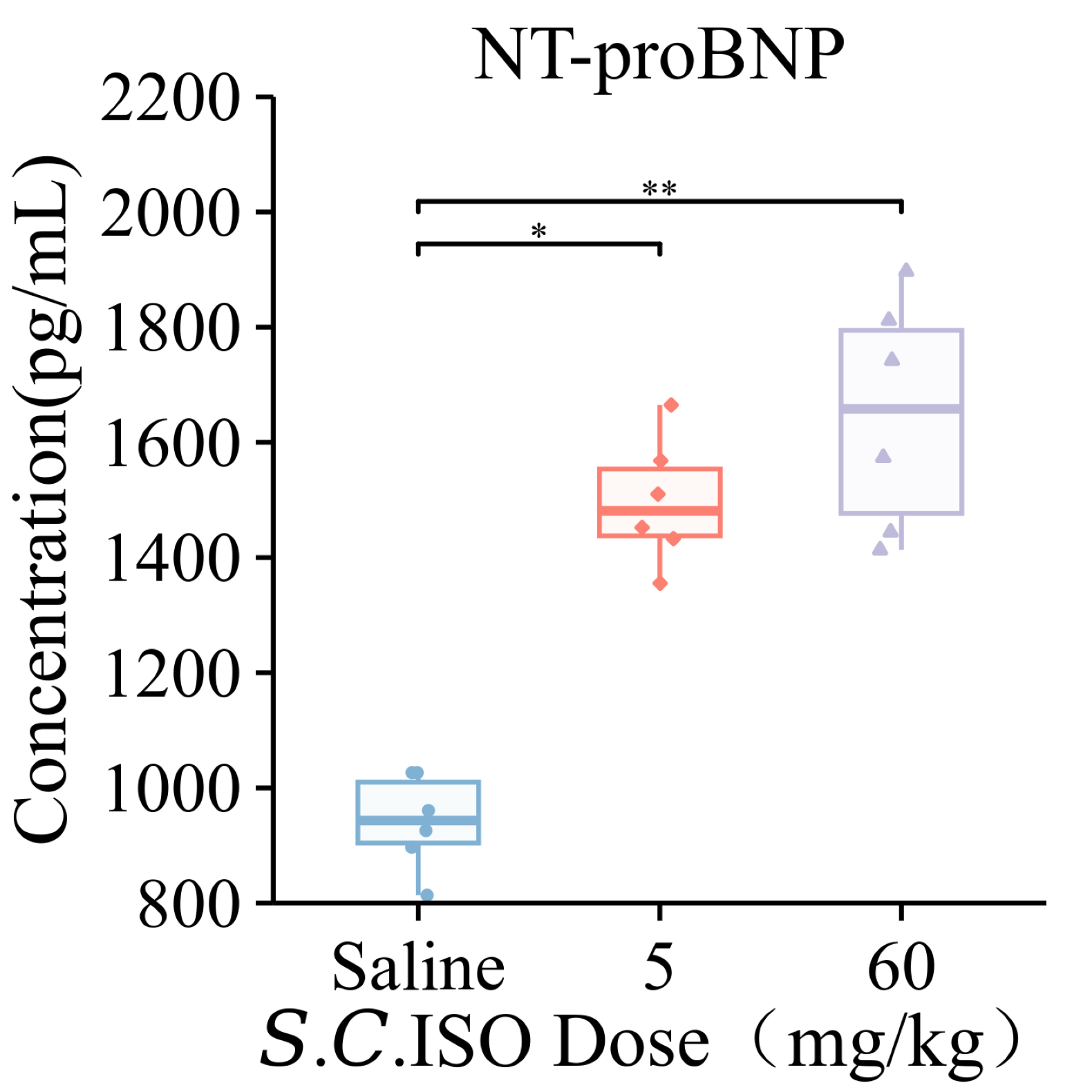

Supplement: S1 File — (ZIP) [file pone.0334880.s001.zip › Supporting information files20251008/Data set for Figure 5/Fig5C-NTpro-BNP/Box chart of NTpro-BNP expression levels in the SC group.tif]
